# A deep learning model for early risk prediction of heart failure with preserved ejection fraction by DNA methylation profiles combined with clinical features

**DOI:** 10.1186/s13148-022-01232-8

**Published:** 2022-01-19

**Authors:** Xuetong Zhao, Yang Sui, Xiuyan Ruan, Xinyue Wang, Kunlun He, Wei Dong, Hongzhu Qu, Xiangdong Fang

**Affiliations:** 1 CAS Key Laboratory of Genome Science and Information, Beijing Key Laboratory of Genome and Precision Medicine Technologies, Beijing Institute of Genomics, Chinese Academy of Sciences/China National Center for Bioinformation, Beijing, 100101 China; 2 University of the Chinese Academy of Sciences, Beijing, 100049 China; 3 Beijing Key Laboratory of Chronic Heart Failure Precision Medicine, Chinese PLA General Hospital, Beijing, 100853 China; 4 Core Laboratory of Translational Medicine, Chinese PLA General Hospital, Beijing, 100853 China; 5 Senior Department of Cardiology, the Sixth Medical Center of PLA General Hospital, Beijing, 100037 China

**Keywords:** Early risk prediction, Deep learning, DNA methylation, Heart failure with preserved ejection fraction

## Abstract

**Background:**

Heart failure with preserved ejection fraction (HFpEF), affected collectively by genetic and environmental factors, is the common subtype of chronic heart failure. Although the available risk assessment methods for HFpEF have achieved some progress, they were based on clinical or genetic features alone. Here, we have developed a deep learning framework, HFmeRisk, using both 5 clinical features and 25 DNA methylation loci to predict the early risk of HFpEF in the Framingham Heart Study Cohort.

**Results:**

The framework incorporates Least Absolute Shrinkage and Selection Operator and Extreme Gradient Boosting-based feature selection, as well as a Factorization-Machine based neural network-based recommender system. Model discrimination and calibration were assessed using the AUC and Hosmer–Lemeshow test. HFmeRisk, including 25 CpGs and 5 clinical features, have achieved the AUC of 0.90 (95% confidence interval 0.88–0.92) and Hosmer–Lemeshow statistic was 6.17 (*P* = 0.632), which outperformed models with clinical characteristics or DNA methylation levels alone, published chronic heart failure risk prediction models and other benchmark machine learning models. Out of them, the DNA methylation levels of two CpGs were significantly correlated with the paired transcriptome levels (*R* < −0.3, *P* < 0.05). Besides, DNA methylation locus in HFmeRisk were associated with intercellular signaling and interaction, amino acid metabolism, transport and activation and the clinical variables were all related with the mechanism of occurrence of HFpEF. Together, these findings give new evidence into the HFmeRisk model.

**Conclusion:**

Our study proposes an early risk assessment framework for HFpEF integrating both clinical and epigenetic features, providing a promising path for clinical decision making.

**Supplementary Information:**

The online version contains supplementary material available at 10.1186/s13148-022-01232-8.

## Background

Chronic heart failure (CHF), characterized by disorders of myocardial energy metabolism and metabolic remodeling, is widely studied by society because of its high morbidity and mortality [1]. It is currently widely accepted that CHF is classified into three subtypes according to the value of left ventricular ejection fraction (LVEF), including heart failure with reduced ejection fraction (HFrEF, LVEF ≤ 40%), heart failure with intermediate ejection fraction (HFmrEF, LVEF > 40% and LVEF ≤ 50%), and heart failure with preserved ejection fraction (HFpEF, LVEF > 50%) [2]. Among them, approximately half of the patients with CHF belong to HFpEF subtype [3]. In a large community-based, longitudinal cohort study of 28,820 participants with 10-year follow-up, the incidence of HFrEF, HFmrEF, and HFpEF was 0.349%, 0.067%, and 0.269% per year, respectively [4, 5]. The all-cause mortality rates of them were 29.5% (15,220/51,496), 26.8% (5402/20,114), and 31.0% (11,681/37,647) in a meta-analysis, respectively [6]. There are no convincing treatments to reduce morbidity or mortality in patients with HFpEF, and only recommendations for management of symptoms and comorbidities [7]. Besides, the diagnosis of HFpEF is challenging because of the normal ejection fraction, which makes it difficult to assess cardiac congestion noninvasively [8, 9]. Therefore, the early prediction of HFpEF may have a beneficial impact on solving health management problems related to HFpEF.

Although risk prediction in CHF has been extensively studied, there are still inadequacies and limitations. Sadiya S. Khan et al. developed a 10-year risk model (included ten clinical risk factors for CHF) but did not discuss the pathogenesis and subtypes of CHF, and the model lacked the ability to learn implicit feature interactions [10]. Benjamin et al. used epigenome-wide association studies to identify epigenetic susceptibility areas associated with CHF but did not consider the clinical characteristics of participants and subtypes of CHF [11]. Some studies focus on small molecule biomarkers, such as natriuretic peptides, microRNAs, inflammatory molecules, cardiac biomarkers, etc., but integrating multiple omics characteristics has received little attention [12]. Epigenetic mechanisms of gene expression have been reported to contribute to the development of cardiovascular diseases and some epigenetic susceptibility regions associated with CHF have been identified, suggesting the potential importance of epigenetic markers for CHF risk prediction [13]. Considering that cardiovascular diseases are regulated by environmental, dietary, and lifestyle factors, epigenetic markers may be more suitable for risk prediction than other omics data (e.g. transcriptomics, proteomics, metabolomics) [14–16]. Recently, DNA methylation has become a promising tool for the study of biomarkers of various cardiovascular diseases [17, 18]. However, risk prediction models integrating clinical characteristics and omic-features for specific subtype of CHF is still lacking. Integrated multiple omics characteristics can provide better risk prediction [19].

The Framingham Heart Study (FHS) cohort is a population-based, multigenerational, and longitudinal cohort study to identify common factors that contribute to cardiovascular disease (https://framinghamheartstudy.org/). It began in 1948 and has undergone six large sampling surveys [20]. The FHS cohort now includes three generations of participants (Original cohort, Offspring cohort, and third generation cohort) and two minority cohorts. The Original cohort of the FHS was recruited from inhabitants of Framingham with random individuals. Study design was based on sampling participants who were free from overt cardiovascular disease. The Framingham Offspring Study, composed of the children of the Original cohort and the spouses of those children. Considering the collection of DNA methylation data, our study used the 8th follow-up of the FHS offspring cohort to determine which biomarkers might be early predictors of HFpEF.

Considering that the interaction between DNA methylation and clinical features may contribute to the early prediction of HFpEF, we proposed an early risk prediction framework for HFpEF by combining multi-omics data interactions through end-to-end machine learning models. The framework fuses Least Absolute Shrinkage and Selection Operator (LASSO) and Extreme Gradient Boosting (XGBoost)-based feature selection, and Factorization-Machine based neural network (DeepFM)-based recommended system to learn the interactions of nonlinear features automatically [21]. Our prediction model provides innovative insights into early risk assessment for HFpEF.

## Methods

### Study population and study design

**Fig. 1:**
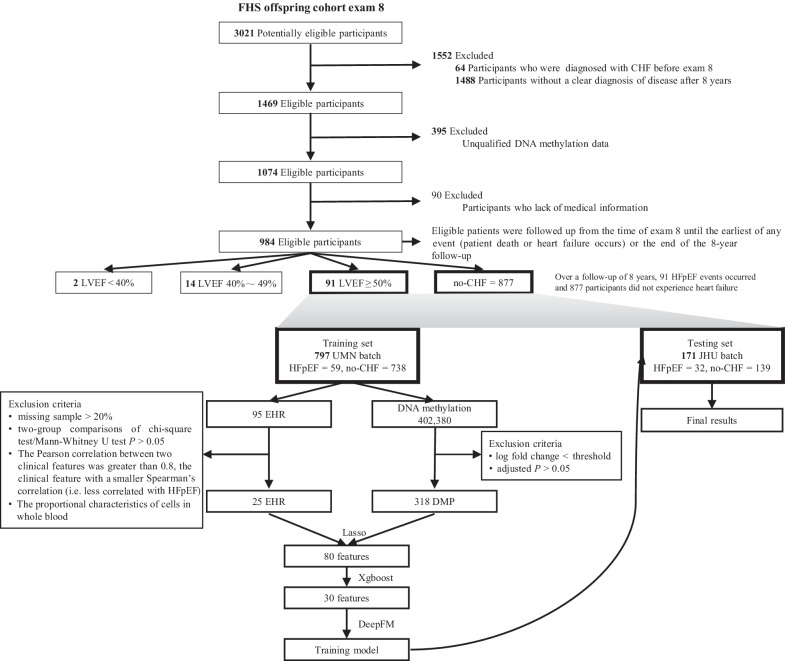
Overview of study population and study design. *FHS* Framingham Heart Study, *UMN* University of Minnesota, *JHU* Johns Hopkins University, *CHF* chronic heart failure, *LVEF* Left ventricular ejection fraction, *HFpEF* heart failure with preserved ejection fraction

Participants who were diagnosed as free of CHF at baseline (the eighth examination cycle, 2005–2008) in FHS Offspring cohort, with a clear disease diagnosis within 8 years (HFpEF or no-CHF), with complete medical information, with qualified DNA methylation data were eligible for inclusion (Fig. 1).

The early prediction observation window was defined as 8 years from baseline. During the 8 years’ follow-up, 91 HFpEF events occurred and 877 participants did not experience heart failure, which is referred to as case–control status. The whole blood samples for DNA methylation, gene expression profile and electronic health record (EHR) data were measured from FHS offspring participants who attended the eighth examination cycle.

### Preprocessing of clinical data

Following thresholds were applied to remove incomplete and non-significant clinical features in training set: missing sample > 20%, two-group comparisons of Chi-square test/Mann–Whitney U test *P* > 0.05. When missing values were less than 20%, missing variables were imputed using nearest neighbor averaging method. If the Spearman’s correlation between two clinical features was greater than 0.8, the clinical feature with a smaller Spearman’s correlation (i.e. less correlated with HFpEF) was discarded ("Blood glucose", "Low-density lipoprotein", "Waist", "Weight"). Detailed information on the removal of clinical features is provided in Materials and Methods Section 1 of the Additional file 1. Continuous clinical features are normalized by scaling between 0 and 1.

### Preprocessing of DNA methylation and gene expression data

Using Infinium HumanMethylation450 BeadChip (Illumina), the methylation level of each cytosine-phosphate-guanine (CpG) locus is represented by the *β*-value, which ranges from 0 (unmethylated) to 1 (fully methylated). DNA methylation array was normalized using the beta mixture quantile dilation algorithm by ChAMP package [22]. DNA methylation was corrected by correcting for sex using the empirical bayes method by SVA package. ChAMP was used to remove all probes located in chromosome X and Y and SNP-related with default parameters. CpG locus missing more than 20% among participants were excluded. Differentially methylated probes (DMPs) were obtained by a linear model using limma package with a criteria of log fold change > threshold (absolute value of fold change plus twice the standard deviation, threshold value = 0.035) and adjusted *P* < 0.05.

In the FHS offspring cohort, whole blood gene expression profiles were obtained from the Affymetrix Human Exon 1.0 ST GeneChip platform. Gene expression microarray data analysis was implemented through linear model fit and empirical bayes statistics for subsequent calculation of Pearson's correlations between gene expression profiles and DNA methylation for paired samples.

### Feature selection for the HFmeRisk model

Feature selection was performed in the training set using LASSO [23] and XGBoost algorithm [24]. For LASSO, the features are filtered according to the area under the ROC curve and misclassification error of different number of features revealed by LASSO, corresponding to "type.measure" parameter "auc" and "class" respectively. tenfold cross-validation is also used for internal validation. "Lambda" is the tuning parameter in the LASSO model used tenfold cross-validation. The R package “glmnet” was used to perform the LASSO.

The R package “xgboost” was used to perform the XGBoost. After parameter adjustment, we finally set the learning rate to 0.5. the minimum loss reduction is 0.5, the maximum depth of the tree is 3, the minimum sum of the required instance weights (Hessian) of the children is 2, and the maximum depth of the tree is 3.

### Factorization-machine based neural networks

This study superimposed DNA methylation and EHR features to form a unique matrix. DeepFM algorithm was used to build an HFpEF risk prediction model. DeepFM extracts DNA methylation and EHR features and learns the hidden feature combinations behind these features [21]. DeepFM jointly trains the overall network in an end-to-end manner, ultimately feeding into the sigmoid function for the early prediction of HFpEF events.

1 $$\hat{y} = {\text{sigmoid}}\left( {y{\text{DNN}} + y{\text{FM}}} \right),$$

where $\hat{y} \in \left( {0,1} \right)$ the predicted HFpEF event, *y*FM is the output of the FM component, and *y*DNN is the output of the deep component. The FM component and deep component are factorization machine and feed-forward neural network, which are used to learn low-order feature interactions and high-order feature interactions. The output of FM is,

2 $$y{\text{FM}} = \left\langle {w,x} \right\rangle + \sum\nolimits_{i = 1}^{d} {\sum\nolimits_{j = i + 1}^{d} {\left\langle {V_{i} ,V_{j} } \right\rangle x_{i} \cdot x_{j} } } ,$$

$w \in R^{d}$ and $V_{i} \in R^{k}$ (k is given) ^2^. The output of DNN is,

3 $$y{\text{DNN}} = W^{\left| H \right| + 1} \cdot a^{\left| H \right|} + b^{\left| H \right| + 1} ,$$

where |*H*| is the number of hidden layers, *a*^(*l*)^ is the output of the embedding layer, *W*^(*l*)^ is the model weight, and *b*^(*l*)^ is the bias of the *l*th layer.

For a given hidden layer in the deep component, we implemented a deep neural network with two hidden layers (256 nodes) using ReLU as the activation function.

4 $$y = f\left( x \right) = {\text{ReLU}}\left( {wx + b} \right)$$

"logloss", which measures the error between the input and the output, was chosen as the objective function. To control overfitting, we added an L2 regularization penalty on the activities of the nodes, and the parameter was set to 0.0001. To optimize the neural network, we used batch normalization and weight decay. The embedding size, batch size and decay were set to 8, 300 and 0.9, respectively. To train the DeepFM algorithm, we used Adam as the optimization algorithm and the learning rate was set to 0.0001, with 400 epochs and 60% dropout. The performance of the DeepFM models was assessed using bootstrapping method. The samples left in the training set will be approximately 63.2% of the original data set, and the remaining samples (36.8%) are used as the validation set [25]. All network models were trained using the TensorFlow framework with TensorFlow 1.15.0 and Python 3.7.3. Calibration of the HFmeRisk was evaluated using the calibration plot of observed versus predicted risk and Hosmer–Lemeshow goodness-of-fit test, where the number of bins to use to calculate quantiles is 10.

### Benchmark model based on machine learning

Nine machine learning algorithms including linear Support Vector Machine, Bagging, Random Forest, RUSBoost, EasyEnsemble, GradientBoosting, XGBoost, LogitBoost, and Mixed Logistic Regression, were used to perform the comparisons. Model parameters are listed in Additional file 1: Materials and Methods Section 2. The performance of the benchmark models was assessed using bootstrapping method.

We evaluated HFmeRisk and the benchmark model using the following criteria: area under the curve (AUC), sensitivity, specificity, and accuracy.

### Decision curve analysis

To estimate the clinical utility of the HFmeRisk model, decision curve analysis (DCA) was performed using the R package rmda to calculate the net benefit of the range of threshold probabilities in the training and testing sets. The threshold probability of DCA is where the expected benefit of prediction is equal to the expected benefit of avoiding prediction. DCA is a trade-off between false positives and false negatives and is mostly used to weigh medical intervention strategies and can be used to screen for beneficiaries and to evaluate the practice value of the model as a whole.

### Biology functional and pathway enrichment analysis

We used the HumanMethylation450 BeadChip array annotation file and Enhancer linking by methylation/expression relationships tool [26] to obtain the genes corresponding to the CpGs loci or the nearest genes at the intergenic region loci in the HFmeRisk model. Gene-based pathway enrichment analysis was performed using ReactomePA and IPA. Gene ontology and pathway analysis of key CpG using the methylation analysis R package missMethyl. Hyper-geometric test was used in gene set pathway analysis.

### Statistical analyses

Two-group comparisons of categorical and continuous variables were performed by using the Chi-square test and the Mann–Whitney U test, respectively. The Pearson’s correlation between CpG and differentially methylated genes (DMGs) is driven mainly by case–control status. Hypergeometric test was used in gene set pathway analysis. In biology functional analyses, the *P* is calculated using a hypergeometric test. All statistical tests were 2-sided, and *P* < 0.05 was considered significant. The adjusted *P* is conducted using Bonferroni corrected. All data analysis and visualization were performed using R 3.5.0 (http://www.r-project.org/) and Python 3.7.3 (https://www.python.org).

## Results

### Characteristics of the study cohorts

The clinical information and DNA methylation data of FHS participants (Offspring Cohort Exam 8) were used to develop a HFpEF risk prediction model. After excluding samples with censoring, with unqualified DNA methylation, and lack of medical information, a total of 984 eligible participants were obtained as the final samples with complete information over a follow up of 8 years (Fig. 1). Among them, 877 participants did not experience heart failure and 91 HFpEF events occurred. A total of 95 EHR variables (the simplified version is shown in Table 1, the full version is shown in Additional file 2: Table S1) and 402,380 CpGs were obtained for further analyses. Since their DNA methylation data were sequenced in University of Minnesota (UMN, 738 no-CHF and 59 HFpEF) and Johns Hopkins University (JHU, 139 no-CHF and 32 HFpEF), respectively, which can be presumed as dependent datasets, data from UMN batch and JHU batch were used as the training set and the testing set (Fig. 1; Table 1). Considering the limited sample size, we did not further balance the sample size. In the training and testing sets, the median follow-up period was 8.69 ± 1.25 years and 8.64 ± 2.05 years, with mean participant’s ages of 64.68 ± 8.29 and 70.13 ± 8.91 years, and the proportion of male participants were 37.39% and 70.76%, respectively (Table 1).

**Table 1:** Demographic of participants in the training set and testing set (the simplified version)

	Training set			Testing set		
	No-CHF (*n* = 738)	HFpEF (*n* = 59)	*P* value	No-CHF (*n* = 139)	HFpEF (*n* = 32)	*P* value
Male	268 (36.3)	30 (50.8)	0.037	100 (71.9)	21 (65.6)	0.62
Age, years	64.0 ± 7.93	73.3 ± 7.84	< 0.001	68.6 ± 8.48	76.8 ± 7.73	< 0.001
Smoking	63 (8.5)	3 (5.1)	0.496	5 (3.6)	2 (6.2)	0.85
BMI, kg/m^2^	27.9 ± 5.20	30.1 ± 5.97	0.004	28.4 ± 4.18	30.5 ± 5.92	0.041
Fasting blood glucose, mg/dL^†^	104 ± 21.8	118 ± 36.7	< 0.001	110 ± 26.7	111 ± 24.8	0.52
LDL cholesterol, mg/dL^†^	112 ± 30.5	100 ± 23.6	0.011	95.2 ± 29.8	87.4 ± 28.4	0.36
HDL cholesterol, mg/dL	58.6 ± 16.9	52.6 ± 16.5	0.007	53.7 ± 14.4	47.2 ± 16.9	0.0087
Average diastolic blood pressure, mmHg	74.3 ± 9.56	71.3 ± 11.3	0.044	73.2 ± 10.2	64.0 ± 10.6	< 0.001
Average systolic blood pressure, mmHg	127 ± 16.6	137 ± 16.9	< 0.001	130 ± 16.5	133 ± 23.7	0.49
Total cholesterol, mg/dL	194 ± 36.4	177 ± 29.9	0.002	170 ± 34.5	159 ± 44.9	0.089
Triglycerides, mg/dL	116 ± 60.1	122 ± 54.9	0.22	109 ± 56.7	115 ± 94.9	0.63
Creatinine serum, mg/dL^†^	0.87 ± 0.20	1.13 ± 0.73	< 0.001	0.986 ± 0.24	1.38 ± 1.13	0.069
Creatinine urine, mg/100 mL^†^	101 ± 60.5	108 ± 56.8	0.25	113 ± 79.4	104 ± 62.3	0.92
Albuminuria urine, mg/L^†^	11.2 ± 38.5	93.0 ± 255	< 0.001	11.6 ± 21.5	116 ± 240	< 0.001
Hemoglobin A1c, whole blood, %	5.66 ± 0.61	5.99 ± 1.19	0.017	5.79 ± 0.844	6.14 ± 0.96	0.011
C reactive protein, mg/L^†^	3.24 ± 6.91	3.82 ± 4.19	0.004	2.06 ± 2.02	5.33 ± 9.62	0.0012
Ejection fraction, % ^†^	66.6 ± 5.14	66.1 ± 6.57	0.85	65.6 ± 5.24	67.3 ± 7.44	0.13
Ventricular rate per minute by ECG, beats/min	62.1 ± 10.0	63.5 ± 10.1	0.22	59.7 ± 9.46	59.7 ± 13.0	0.85
Atrial fibrillation	14 (1.9)	6 (10.2)	< 0.001	16 (11.5)	21 (65.6)	< 0.001
Stroke	2 (0.3)	1 (1.7)	0.54	15 (10.8)	7 (21.9)	0.16
Left ventricular hypertrophy^†^	5 (0.7)	2 (3.4)	0.15	0 (0)	0 (0)	–
Atrial enlargement^†^	8 (1.1)	4 (6.8)	0.003	6 (4.3)	2 (6.2)	1
Coronary heart disease	17 (2.3)	9 (15.3)	< 0.001	45 (32.4)	16 (50.0)	0.095
Myocardial infarction	3 (0.4)	0 (0)	1	24 (17.3)	7 (21.9)	0.72
Right ventricular hypertrophy^‡^	0 (0)	0 (0)	–	0 (0)	0 (0)	–
Aspirin	239 (32.4)	31 (52.5)	0.003	89 (64.0)	21 (65.6)	1
Folic acid	30 (4.1)	6 (10.2)	0.065	11 (7.9)	4 (12.5)	0.631
Statin	220 (29.8)	24 (40.7)	0.11	94 (67.6)	21 (65.6)	0.993
Thiazides	86 (11.7)	9 (15.3)	0.54	22 (15.8)	7 (21.9)	0.575
Diuretics	17 (2.3)	12 (20.3)	< 0.001	4 (2.9)	10 (31.2)	< 0.001
Potassium	21 (2.8)	2 (3.4)	1	2 (1.4)	0 (0)	1
Aldosterone	6 (0.8)	1 (1.7)	1	10 (7.2)	4 (12.5)	0.529
Amiodarone	2 (0.3)	0 (0)	1	0 (0)	0 (0)	–
Omega 3	73 (9.9)	4 (6.8)	0.583	24 (17.3)	3 (9.4)	0.404
Vasodilators	6 (0.8)	1 (1.7)	1	10 (7.2)	4 (12.5)	0.529
Co-Q 10	18 (2.4)	1 (1.7)	1	4 (2.9)	1 (3.1)	1
ß-blocker	128 (17.3)	23 (39.0)	< 0.001	61 (43.9)	20 (62.5)	0.0882
Angiotensin II antagonists	41 (5.6)	10 (16.9)	0.002	12 (8.6)	5 (15.6)	0.388
ACEI	133 (18.0)	19 (32.2)	0.013	52 (37.4)	15 (46.9)	0.431
Warfarin	13 (1.8)	3 (5.1)	0.204	4 (2.9)	3 (9.4)	0.239
Clopidogrel	4 (0.5)	1 (1.7)	0.824	9 (6.5)	6 (18.8)	0.062

Categorical variables and continuous variables with Chi-square test and Mann–Whitney *U* test were used for two-group comparison

Values are mean ± SD or *n* (%). *P* value is the comparison of heart failure patients versus non-heart failure controls

*CHF* chronic heart failure, *HFpEF* heart failure with preserved ejection fraction, *LDL* low density lipoprotein, *HDL* high density lipoprotein, *ACEI* angiotensin-converting enzyme inhibitor

^†^Missing sample less than 20%. ‡ Missing sample more than 20%

### Prediction model construction using DeepFM

**Fig. 2:**
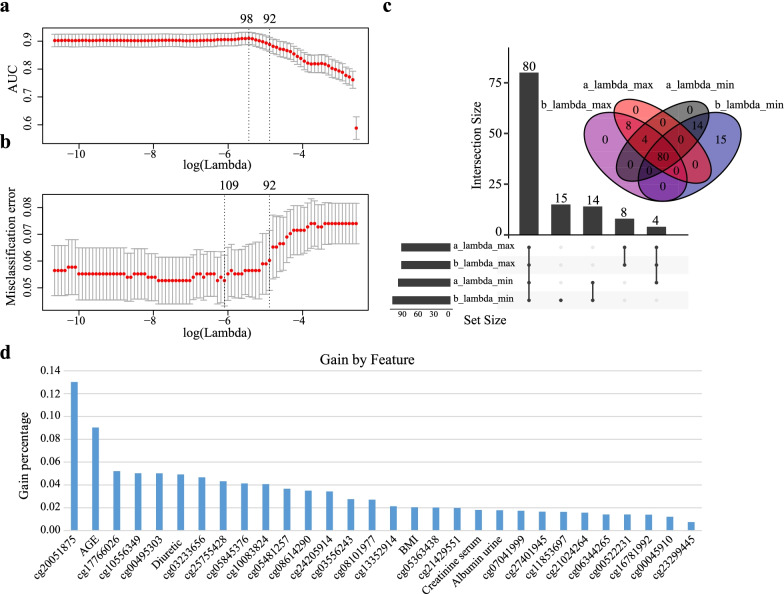
30 features obtained by LASSO and XGBoost algorithms. **a** AUC with different number of characteristics as revealed by the LASSO model. **b** Misclassification error for different number of features revealed by the LASSO model. In **a** and **b**, the grey lines represent the standard error and the vertical dotted lines represent optimal values by minimum criteria (left) and the largest value of lambda such that the error is within one standard error of the minimum (right). The upper abscissa is the number of non-zero coefficients in the model at this time and the lower abscissa is log Lambda, which is the tuning parameter used for tenfold cross-validation in the LASSO model. **c** The intersection of non-zero coefficients in **a** and **b**. 80 non-zero coefficients are obtained in the LASSO model. **d** The best model features were ranked based on the gain index in xgboost model. The xgboost model further simplified the 80 features from the LASSO model, and finally, 30 valid features were obtained. The gain index represents the fractional contribution of each feature to the model based on the total gain of this feature’s splits

After data pre-processing, we obtained 318 DMPs and 25 clinical characteristics (Additional file 2: Table S2). Next, we performed feature selection using LASSO and XGBoost algorithms. The LASSO algorithm simultaneously performs feature selection and regularization, aiming to enhance the predictive accuracy and interpretability of statistical models by selectively putting variables into the model. The important parameter, lambda, contributes to feature selection. We obtained 4 set of features according to the value of lambda (lambda.min and lambda.1se for calculating AUC and misclassification error) and obtained 80 features intersected (Fig. 2a–c). The XGBoost algorithm integrates many weak classifiers together with regularized boosting technique to form a strong classifier. It took 80 features from LASSO and further reduced to 30 features, including 5 clinical variables and 25 CpG loci, which were next fed into the DeepFM model. Five clinical variables (age, diuretic use, body mass index (BMI), albuminuria, and serum creatinine) accounted for nearly 20% of the contribution, explained by the gain index (Fig. 2d). The cg20051875 had the largest gain index, accounting for 13% of the total contribution. In addition, 25 CpGs accounted for 80% of the total contribution, although the contribution of each CpG was weak.

**Fig. 3:**
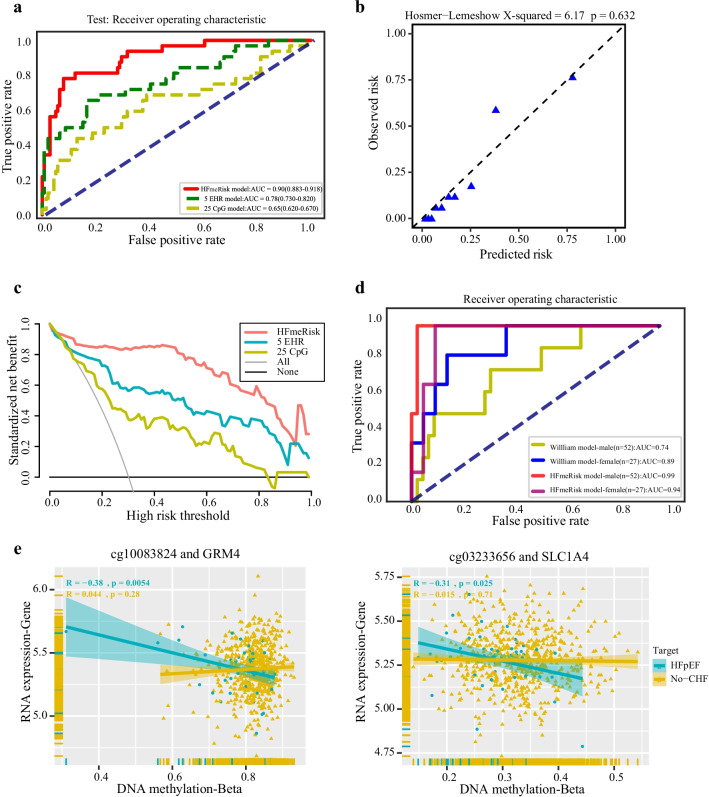
Performance of the HFmeRisk model. **a** AUC results of the prediction performance according to different features in the testing set. “(HFmeRisk/EHR/CpG model)” indicates the model with EHR and DNA methylation data, the model with DNA methylation data only, and the model with EHR data only, respectively. **b** Calibration plot of the DeepFM model in the testing set using 30 features. The Hosmer–Lemeshow statistic was 6.17, with *P* = 0.632. **c** Decision curve analyses of the HFmeRisk, 5 EHR model risk and 25 CpGs model risk in the testing cohort. **d** AUC results for the HFmeRisk model versus the Willliam’s model in male/female participants. **e** The association of CpG (cg10083824/cg03233656) and its DMG expression (GRM4/SLC1A4) in blood samples of FHS participants. *X*-axis is beta value of DNA methylation, *Y*-axis is expression value of RNA data. Rug plots display individual cases in *X*- and *Y*-axis. The smooth curve shows linear smooths in case/control status. The Pearson's correlation between CpG and DMG is driven mainly by case–control status. DMG, differentially methylated gene. The triangle represents the no-CHF participants; the dot represents the HFpEF participants

Based on the DeepFM method, we developed the HFmeRisk model to investigate the feasibility of the early-stage risk prediction for HFpEF using 25 DNA methylation sites and 5 clinical features. We also tested the performance of the DeepFM algorithm using only 5 clinical features or 25 DNA methylation features alone. In the testing set, the AUCs for the HFmeRisk model, the model with EHR alone, and the model with CpGs alone were 0.90 (95% confidence interval [CI] 0.88–0.92), 0.78 (95% CI 0.73–0.82), and 0.65 (95% CI 0.62–0.67), respectively (Fig. 3a; Additional file 2: Table S3). Although the DNA methylation model achieved a lower AUC, AUC was improved when combined with EHR to form the HFmeRisk model. In summary, the “EHR + DNA methylation” model achieved the best AUC in most cases in the testing set.

Calibration of the HFmeRisk model is shown in Fig. 3b. The Hosmer–Lemeshow statistic was 6.17, with *P* = 0.632, indicating that the HFmeRisk model is well calibrated in the testing set.

Similarly, using the decision curve (Fig. 3c), the HFmeRisk model also showed a higher net benefit than the other models. Decision curve of HFmeRisk model is higher than the gray (“All”) and black (“None”) line. Patients would benefit more from the prediction of HFmeRisk model compared to other schemes (5 EHR model and 25 CpGs model) in most ranges.

### Evaluation of the HFmeRisk

We evaluated the performance of HFmeRisk from the aspect of number of features, effect of age, external data verification, comparison with other models, comparison with other omics features, and covariate shift between training and testing subjects, respectively. To evaluate the effect of the number of features on the HFmeRisk model, we selected the top 5, top 10 and top 15 features for further modeling and found that the number of features had a strong effect on the model results (Additional file 2: Table S4). These results suggest that the number of features in the model cannot be reduced further so as to maintain sufficient predictive performance.

Since age is a very critical clinical characteristic in the prediction of HFpEF, it is particularly important to assess the impact of aging-related CpGs on the HFmeRisk model [27, 28]. We used aging-related CpGs reported in 3 articles [29–31] to validate their predictive power, and obtained AUC of 0.655, 0.530, and 0.534 in the testing set, respectively (Additional file 1: Materials and Methods Section 3 and Additional file 2: Table S5), indicating that the 26 age-related CpGs mentioned in Hannum G et al*.* study appeared to have equal predictive power to the 25 CpGs in the HFmeRisk model (AUC = 0.65). However, we combined 26 age-related CpGs mentioned in Hannum G et al*.* study and 5 clinical features of HFmeRisk model (age, diuretic use, BMI, albuminuria, and serum creatinine) together and obtained AUC of 0.858 in the testing set (Additional file 2: Table S5) which is less than that in HFmeRisk model (AUC = 0.90), indicating that the HFmeRisk model performed better in the testing set from the combined feature perspective. The reason may be that the 5 clinical variables we considered already included age, although the age-related 26 CpGs and the 25 CpGs in the HFmeRisk model had comparable predictive power, the age-related CpGs showed no advantage when combined with the clinical characteristics (including age). Also, using only clinical characteristics (age and the remaining four clinical variables) performed worse than the HFmeRisk model. After that, we also did a Pearson correlation analysis between 25 CpGs and age in the training and testing set, and the absolute value of the correlation was less than 0.24 (Additional file 2: Table S6). In addition, when we performed the HFpEF prediction using the age feature alone, the AUC is 0.68 (Additional file 2: Table S5), which further confirms that age has some predictive power, but it does not predict HFpEF well alone.

To evaluate the impact of the sample size of training set on the HFmeRisk model, we randomly selected 25%, 50%, 60%, and 75% of the training set participants and found that the results of the testing set performed stably regardless of the sample size of the training set, indicating that the prediction results were independent of the sample size of the training set (Additional file 2: Table S7).

Because DNA methylation data is not currently available in prospective cohort populations and the HFmeRisk model contains five clinical features, there are currently no suitable datasets in public databases that could be used as external testing sets. To further illustrate the validity of the HFmeRisk model, we evaluated the model using 36 patients who had developed HFpEF and 2 samples who did not have HFpEF after 8 years in the Framingham Heart Study cohort but did not appear in the HFmeRisk model, and obtained an AUC of 0.82 (Additional file 3: Fig. S1). We attempted to demonstrate that the predictive power of the HFmeRisk model for HFpEF is reliable by evaluating 38 samples.

In addition, we compared the performance of the HFmeRisk model with nine benchmark machine learning models that are currently widely used (Additional file 1: Materials and Methods Section 2). Although there were slight differences among their AUCs (AUC = 0.63–0.83) using the same 30 features, the DeepFM model still achieved the best performance (AUC = 0.90, Additional file 3: Fig. S2 and Additional file 2: Table S3). We also used the Cox regression model, a common model for disease risk prediction, for comparison with machine learning model. If the variables with *P* < 0.05 in univariate analysis were used for multivariate analysis, the screening of variables from the 450 K DNA microarray data works tremendously, so we directly used the 30-dimensional features obtained by dimensionality reduction for multivariate analysis of cox regression. The performance of the models was compared using the C statistic or AUC, and the DeepFM model (AUC = 0.90) performed better than the Cox regression model (C statistic = 0.85). Calibration was also assessed by comparing predicted and observed risk (Hosmer–Lemeshow *P* = 0.199). The calibration curves for the possibility of 8-year early risk prediction of HFpEF displayed obvious concordance between the predicted and observed results (Additional file 3: Fig. S3).

To assess whether other omics data could also predict HFpEF, HFmeRisk was compared with other omics models (“EHR + RNA” model and “EHR + microRNA” model). For “EHR + RNA” model and “EHR + microRNA” model, we used the consistent feature selection and modeling approach with the HFmeRisk model (Additional file 1: Materials and Methods Sections 4 and 5; Additional file 3: Fig. S4–S9). The AUC results show that the HFmeRisk model combining DNA methylation and EHR has the best performance under current conditions compared to the "EHR + RNA" model (AUC = 0.784; Additional file 3: Fig. S6) and "EHR + microRNA" model (AUC = 0.798; Additional file 3: Fig. S9), suggesting that DNA methylation is suitable to predict the CHF risk than RNA.

To test whether the training subjects and the testing subjects are sufficiently similar in terms of clinical parameters, which is equivalent to determine whether a covariate shift has occurred, we used adversarial validation to test whether the distribution of the training and testing sets are consistent. If a covariate shift occurs in the data, it is theoretically possible to distinguish the training data from the testing data with a higher accuracy by a classifier. Here, AUC and Matthews correlation coefficient (MCC) were used to measure the results [32]. The general MCC threshold can be set to 0.2, and MCC > 0.2 indicates the phenomenon of covariate shift. The MCC of training and testing subjects is 0.105 and the AUC is 0.514 (Additional file 1: Materials and Methods Section 6; Additional file 3: Fig. S10), indicating that no covariate shift occurs and the training set and the testing set are distributed in the same way.

### HFmeRisk model is superior to the published CHF risk prediction model

Furthermore, we compared the performance of the HFmeRisk model with that of published CHF risk prediction models. William B. Kannel et al*.* proposed a 4-year risk appraisal model (using 9 EHR features) to assess the risk of CHF by gender in the FHS cohort using a mixed logistic regression algorithm [33]. Since we use the same FHS cohort to build models, it is possible to evaluate both models simultaneously. Due to data limitations, the reconstructed Willliam’s model contains only 79 participants (52 males and 27 females). Detailed characteristic information is listed in Additional file 1: Materials and Methods Section 7. Ultimately, the AUCs for the HFmeRisk model and Willliam’s model were 0.99 and 0.74 for male, 0.94 and 0.89 for female, respectively (Fig. 3d). In the HFmeRisk model, the number of male and female participants are different but the AUC results are similar, which shows that the model is not sensitive to gender. Additionally, adding the gender feature to the HFmeRisk model did not get an improvement in the testing set (Additional file 2: Table S8). Since our data did not include the characteristics of other published articles, we directly compared the AUC or C statistic of the two published articles. Sadiya S. Khan et al*.* described 10-year risk equations for CHF (using 10 EHR features) with a C-statistic of 0.71–0.87 in the validation set, and Edward Choi et al. established an early detection model (using 58,652,000 medical codes) of CHF with an AUC < 0.88 in the testing set [10, 34]. Their AUCs are all less than that of HFmeRisk, indicating the superiority of risk prediction by both DNA methylation and clinical features.

### Biological functions of CpGs involved in HFmeRisk model

**Fig. 4:**
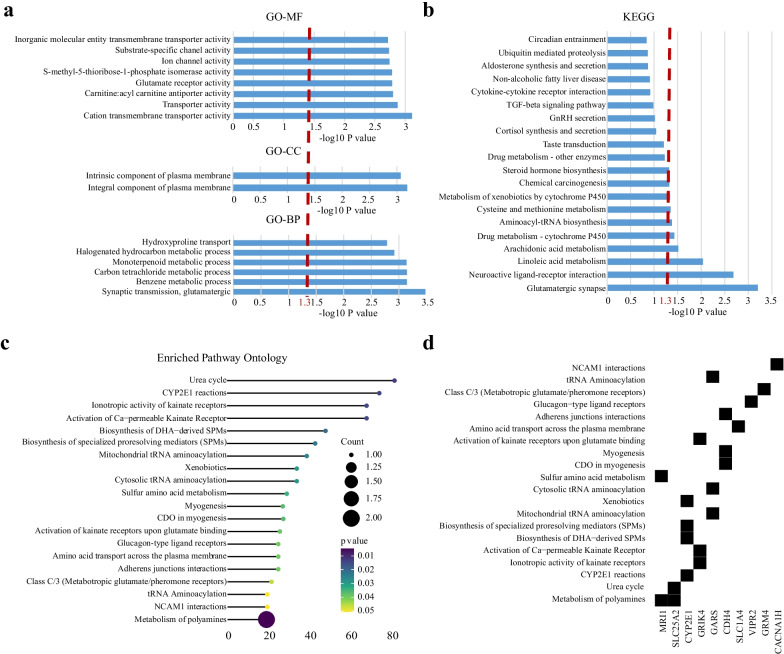
Gene ontology categories and pathways analysis of DNA methylation loci in HFmeRisk model. **a** Gene ontology enrichment of CpG loci (*MF* molecular function, *CC* cellular component, *BP* biological process). **b** KEGG pathways enrichment of CpG loci. In **a** and **b**, the red line is where the − log10 *P* values = 1.3 (*P* = 0.05). **c** Using ReactomePA, pathways are sorted based on the fold enrichment (*x*-axis). Fold enrichment was defined as the ratio of two proportions, the gene ratio and the BG ratio; Gene ratio indicates the number of genes annotated to a pathway within the specific list of differential genes among the major contributors that are included in the database; BG ratio denotes the total number of genes in the gene set and the total number of all genes in any gene set. The size of the dot indicates the number of genes that are annotated to the pathway, and the color of the dot indicates the *P* values. From these values, the raw “*P* values” is calculated using a hypergeometric test. **d** Correspondence between genes and pathways in **c**

Next, we investigated the biological function of the 25 CpGs in HFmeRisk model. Approximately 2/5 of them were located in the promoter region (TSS200, TSS1500, 5UTR, and 1stExon). Most of the CpG loci were located in CpG islands or the “Open sea” and located on 17 genes and 8 intergenic regions in total (Table 2). Among them, the DNA methylation level of cg10083824 and cg03233656 significantly negatively associated with the expression of target genes, *GRM4* (*R* =  −0.38, *p* = 0.0054) and *SLC1A4* (*R* = −0.31, *p* = 0.025), respectively, in HFpEF participants, while the association among normal participants were not obvious (Fig. 3e). It implies that the existence of some regulatory role of DNA methylation and gene expression. They were involved in 16 gene ontology terms (Fig. 4a; Additional file 2: Table S9) and 10 KEGG pathways (Fig. 4b; Additional file 2: Table S10). Overall, they have key functions for intercellular signaling, interaction and energy metabolism, and involved in pathways of urea cycle (*SLC25A2*/cg05845376) [35], the synthesis of cytochrome enzymes (*CYP2E1*/cg21024264) [36], the amino acid metabolism (*MRI1*/cg25755428, *GRM4*/cg10083824, and *GRIK4*/cg06344265) [37], the amino acid transportation (*SLC1A4*/cg03233656) [38], the activation of the amino acid (*GARS*/cg21429551) [39] (Fig. 4c, d; Additional file 2: Table S11–S12; Additional file 3: Fig. S11). Together, these findings give new evidence into the HFmeRisk model.

**Table 2:** The 25 CpGs associated with HFmeRisk model

Probe	Chr	Position	Closest gene	Distance to gene	Side	UCSC RefGene Group	Relation to UCSC CpG Island	Enhancer
cg00045910	chr10	23,466,070	PTF1A	15,184	R	IGR	S Shelf	NA
cg00495303	chr18	3,771,110	DLGAP1	0	–	Body	N Shore	NA
cg00522231	Chr2	9,549,277	ITGB1BP1	0	–	Body	Open sea	NA
cg03233656	chr2	65,214,625	SLC1A4	0	–	TSS1500	N Shore	NA
cg03556243	Chr3	114,343,779	ZBTB20	0	–	5'UTR;1stExon;TSS1500	Open sea	NA
cg05363438	chr1	224,301,382	FBXO28	0	–	TSS1500	N Shore	NA
cg05481257	chr2	20,870,211	GDF7	0	–	Body	Island	NA
cg05845376	chr5	140,683,632	SLC25A2	0	–	TSS200	Island	NA
cg06344265	chr11	120,530,973	GRIK4	0	–	TSS200	Open sea	NA
cg07041999	chr8	2,178,272	MYOM2	− 64,796	L	IGR	Open sea	NA
cg08101977	chr16	1,231,407	CACNA1H	0	–	Body	S Shore	NA
cg08614290	chr7	158,938,491	VIPR2	0	–	TSS1500	Island	NA
cg10083824	chr6	34,102,147	GRM4	0	–	TSS1500	Open sea	NA
cg10556349	chr10	835,070	DIP2C	− 99,386	L	IGR	Open sea	NA
cg11853697	chr20	60,510,235	CDH4	0	–	Body	N Shore	TRUE
cg13352914	chr1	63,760,405	FOXD3	28,323	R	IGR	Open sea	TRUE
cg16781992	chr4	20,985,623	KCNIP4	0	–	Body;5'UTR	Open sea	NA
cg17766026	chr10	102,405,781	HIF1AN	− 86,025	L	IGR	Open sea	TRUE
cg20051875	chr12	68,201,286	DYRK2	− 142,099	L	IGR	Open sea	TRUE
cg21024264	chr10	135,341,025	CYP2E1	0	–	1stExon	N Shore	NA
cg21429551	chr7	30,635,762	GARS	0	–	Body	S Shore	NA
cg23299445	chr15	73,113,226	ADPGK	− 35,038	L	IGR	Open sea	TRUE
cg24205914	chr10	62,761,575	RHOBTB1	0	–	TSS1500	Island	NA
cg25755428	chr19	13,875,111	MRI1	0	–	TSS1500	Island	NA
cg27401945	chr10	118,919,088	VAX1	− 21,275	L	IGR	N Shelf	TRUE

Furthermore, we explored the relationship of the genes twenty-five CpGs located with disease or trait by intersecting with published GWAS results. All these genes were reported to be associated with risk factors for heart failure such as BMI (*GRM4*, *SLC25A2*, and *ZBTB20*) [40], systolic blood pressure (*SLC1A4*, *ZBTB20*, and *SLC25A2*) [41], ejection fraction (*SLC1A4* and *DLGAP1*) [42], atrial fibrillation (*SLC25A2* and *SLC1A4*) [43], coronary artery disease (*ZBTB20* and *SLC25A2*) [44], type 2 diabetes (*ZBTB20*) [45], cardiac Troponin-T levels (*DLGAP1*) [46], diastolic blood pressure (*RHOBTB1*) [47], gout (*CYP2E1*) [48], implying the scientific validity of CpGs in model for CHF risk prediction.

## Discussion

In this report, we established and validated the HFpEF early risk prediction model HFmeRisk using the FHS cohort. HFmeRisk evaluated the early risk prediction of HFpEF from an epigenetic perspective (25 CpGs have key functions in intercellular signaling, interaction and energy metabolism) and environmental exposures perspective (age, diuretic use, body mass index, albuminuria, and serum creatinine). The HFmeRisk model demonstrated excellent discriminatory and calibration power in the early risk of HFpEF with an AUC of 0.90 (95% CI 0.89–0.90) and Hosmer–Lemeshow statistic was 6.17, with *P* = 0.632 in the testing set. HFmeRisk leverages the recommendation system-based deepFM algorithm and feature selection-based lasso and xgboost algorithms, and learns the hidden feature combinations behind these features to provide innovative insights into early risk assessment for HFpEF. The HFmeRisk model provides a implications to further facilitate guiding clinical risk assessment at the individual level.

It is worth noting that the HFmeRisk model (EHR + DNA methylation) outperforms the “EHR only” and “DNA methylation only” models, which supports the contribution of epigenetics to the early diagnosis model of HFpEF, and the addition of epigenetic features allows the prediction model to achieve better prediction, confirming that DNA methylation provides innovative ideas for further research on the development of HFpEF [49].The HFmeRisk model proposed in this study for the early assessment of HFpEF was superior to previously published models, e.g. Willliam et al*.*, Sadiya S. Khan et al*.* and Edward Choi et al. model [10, 33, 34]. These models achieved good predictions from the perspective of focusing on clinical characteristics. However, considering that they did not focus on different subtypes, did not focus on omics data, and did not consider the interaction between different types of features, the HFmeRisk model achieved a small breakthrough. We also compared the performance of the mixed logistic regression model (from the study of William B. Kannel et al*.*) and the DeepFM model of this study. Although the mixed logistic regression model performed well in terms of AUC (AUC = 0.83), the DeepFM model still achieved the best performance (AUC = 0.90).

Additionally, 25 CpGs in the HFmeRisk model have key functions related to intercellular signaling, interaction and energy metabolism. This may suggest that intercellular signaling, interaction and energy metabolism were subjected to epigenetic regulation and were involved in driving lesion progression and the development of HFpEF. These results may provide clues to pathways related to the regulation of heart failure development by 25 DNA methylation loci. Five clinical variables included in the HFmeRisk model, including age, diuretic use, BMI, albuminuria, and serum creatinine, were all closely related to the heart failure. It is well known that HFpEF and age are closely related, the risk of HFpEF increases sharply with age [3]. Similarly, through direct and indirect effects, an increase in BMI is strongly associated with the development of HFpEF [50]. In addition, elevated blood creatinine levels, usually a sign of chronic renal failure, may also cause HFpEF [51]. Albuminuria is a significant predictor of worse outcomes and cardiovascular hospitalization [52]. The function of CpGs and relationship of clinical features with heart failure further support the validity of feature selection in our model.

We also obtained the significantly correlation between 2 CpGs and its DMGs expression levels. Because DNA methylation and clinical features can describe disease states in different dimensions, they may be internally correlated. In addition, Framingham Offspring cohort contains not only DNA methylation data, but also RNA and microRNA data, and we have done the similar analysis separately. Both the "EHR + RNA" model and the "EHR + microRNA" model showed less good results than the HFmeRisk model. MicroRNAs have been reported to have some predictive value for HFpEF [53], offering attractive potential as epigenetic disease biomarkers. Unfortunately, in the present dataset, microRNAs are severely missing, otherwise the effect of microRNAs on HFpEF early diagnosis model would also be uncovered.

The most important feature of the DeepFM algorithm is its ability to learn the hidden feature combinations behind the input features. Simple feature stitching cannot achieve the deep integration of internal features, so the DeepFM model is very reasonable for the integration of multiomics data. We also showed that the DeepFM model performs better than the benchmark machine learning models. The bootstrapping method used in this study is uniform sampling with put-back from a given training set, which provides a good idea for solving the small subsample test evaluation problem.

In addition, a comprehensive evaluation of the HFmeRisk model is presented in this paper. The sufficient predictive performance of HFmeRisk was demonstrated by evaluating the number of features, and the sample size of the training set. The predictive power of age was demonstrated by testing age-related DNA methylation sites, but age-related DNA methylation sites did not give better results than HFmeRisk. Adversarial validation was used to test whether the distributions of the training and testing sets were consistent to assess whether the training and testing subjects were sufficiently similar in terms of clinical parameters.

In the future, we will consider both biological mechanism validation and model optimization. In aspect of biological mechanism research, we will consider adding other data, such as family information, transcriptomic and genetic data, to find the real reason why DNA methylation acts as a predictor from the perspective of expression Quantitative Trait Loci and methylation Quantitative Trait Loci analyses which will contribute to mechanisms of disease pathophysiology, and to provide evidence for functional effects for HFpEF and insight into genetic mediated epigenetic response mechanisms that modulate epigenetic effects in the whole blood and risk for HFpEF. We also will focus on epigenomic and enhancer-gene remote interactions yields new perspectives on disease-associated loci, which will also be important for understanding the dynamic interplay between epigenome in HFpEF. Another, considering that mechanisms such as fibrosis and inflammation are involved in the development of heart failure, single-cell transcriptome mapping of non-myocytes and leukocytes in the heart of adult heart failure patients is obtained using single-cell transcriptome sequencing data, which will provide theoretical basis for predictive models and new therapeutic approaches for HFpEF patients [54]. In aspect of model optimization, we consider the inclusion of a larger external test sample to improve the credibility of the model. In addition, if more samples of other races are collected to be able to really propose a corresponding prediction model for different races. In the future, we expect to have developed a calculator that will allow clinicians to automatically calculate a patient's risk of HFpEF as a reference in the clinical decision making process.

## Conclusions

CHF is a severe or advanced manifestation of various cardiac conditions with high mortality and readmission rates [55]. Therefore, it is important to have individualized risk estimates to assist in further management decisions for HFpEF. Here, we constructed the HFmeRisk model starting from the existing markers (DNA methylation and EHR) to find clues for the occurrence of HFpEF from the perspective of pathogenesis, which provides some guidance for the early risk prediction of HFpEF. These results indicate that DNA methylation and clinical features may provide empirical information on the occurrence of HFpEF, thus providing a promising path for clinical decision making.

### Limitations

Our research still has some limitations. We analyzed DNA methylation and RNA expression in whole blood, which may differ from the values in heart tissue samples. However, it is unrealistic to obtain tissue samples in the prospective study. In the future, we will consider providing proof of consistency between blood and target tissue results. The current study does not consider the censoring problem, and the censored samples should be retained as much as possible. In the future research, we will further explore the censoring problem, such as how to deal with the censoring problem in machine learning modeling, etc. The FHS cohort includes Caucasians and a small number of East Asians, and it is unclear whether we can reproduce our conclusions in other races. Due to the limited sample size of the training and testing sets, the reliability of the results is questioned to some extent. In addition, there is no suitable external testing set to demonstrate the effectiveness of the HFmeRisk model due to the difficulty in obtaining DNA methylation data for prospective cohorts. We sought to demonstrate that the predictive power of the HFmeRisk model for HFpEF is reliable using 38 Framingham Heart Study cohort participants who did not appear in the HFmeRisk model as a simulation test, which also appeared to demonstrate sufficient predictive power of HFmeRisk (AUC = 0.82). DNA methylation was only collected at exam 8, so we were unable to evaluate longitudinal changes. The applicability of DNA methylation arrays in extensive screening may be limited by cost. After in-depth characterization screening, sequencing may have the greatest advantages because it is cost-effective and can be used to calculate the risks for other diseases. Compared with clinical characteristics, DNA methylation is less effective in enhancing the model. For the analysis of DNA methylation, the results of differentially methylated region and differentially methylated block analysis can be considered subsequently, and large segments of methylation regions are more convincing than single methylation sites. In addition, although AUC is currently considered to be a standard approach for assessing the accuracy of predictive distribution models, it also has limitations. AUC is a trade-off between true positive rate and false positive rate, and AUC has limited clinically meaningful to clinicians [56]. Finally, due to small sample size and insufficient statistical power, we did not analyze additional heart failure subtypes (HFmrEF and HFrEF). But the same idea can be put into practice in other diseases, providing innovative insights to further guide clinical risk assessment at the individual level and providing a promising pathway for clinical decision making.

## Supplementary Information

**Additional file 1.** Supplementary materials and methods.

**Additional file 2.** Supplementary tables.

**Additional file 3.** Supplementary figures.

## Data Availability

The data that support the findings of this study are available from dbGaP website but they were restrictedly applied with a license for the current study. Therefore, these data are not publicly available. The FHS cohort were obtained from dbGaP website (accession: phs000007.v30.p11). This study used DNA methylation data from phs000724.v7.p11, transcriptome mRNA and microRNA data from phs000363.v17.p11 on the dbGaP website.

## Abbreviations

HFpEF: Heart failure with preserved ejection fraction
CHF: Chronic heart failure
LVEF: Left ventricular ejection fraction
HFrEF: Heart failure with reduced ejection fraction
HFmrEF: Heart failure with intermediate ejection fraction
FHS: Framingham Heart Study
LASSO: Least Absolute Shrinkage and Selection Operator
XGBoost: Extreme Gradient Boosting
DeepFM: Factorization-Machine based neural network
CpG: Cytosine-phosphate-guanine
DMPs: Differentially methylated probes
DCA: Decision curve analysis
DMGs: Differentially methylated genes
BMI: Body mass index
AUC: Area under the curve
EHR: Electronic health record
MCC: Matthews correlation coefficient
UMN: University of Minnesota
JHU: Johns Hopkins University
CI: Confidence interval
