# Supplementary material for: A deep learning model for early risk prediction of heart failure with preserved ejection fraction by DNA methylation profiles combined with clinical features
Source: Clin Epigenetics. 2022 Jan 19;14:11. doi: 10.1186/s13148-022-01232-8 (PMC8772140; doi:10.1186/s13148-022-01232-8)
Supplement: Supplementary file 1 — Additional file 1. Supplementary materials and methods. [file 13148_2022_1232_MOESM1_ESM.docx]

Supplementary Online Content

[Supplementary Materials and Methods 2](#_Toc93331717)

[Section 1: Detailed information on the exclusion of clinical features. 2](#_Toc93331718)

[Section 2: Parameter settings of the benchmark model. 3](#_Toc93331719)

[Section 3: Aging related CpGs in aging-related articles. 4](#_Toc93331720)

[Section 4: “EHR+ RNA” model. 4](#_Toc93331721)

[Section 5: “EHR+ mircroRNA” model. 5](#_Toc93331722)

[Section 6: The Matthews correlation coefficient. 5](#_Toc93331723)

[Section 7: Parameter settings of the Willliam’s model. 5](#_Toc93331724)

[Reference 7](#_Toc93331725)

[Abbreviations 7](#_Toc93331726)

# Supplementary Materials and Methods

## Section 1: Detailed information on the exclusion of clinical features.

The following thresholds were applied to remove incomplete clinical features (missing sample > 20%), “diabetes” and “Right ventricular hypertrophy” be removed.

The following thresholds were applied to remove nonsignificant clinical features (two-group comparisons of chi-square test/Mann-Whitney U test *P* > 0.05), "Ejection fraction", "Omega 3", "Statin", "Thiazides", "Potassium", "Aldosterone", "Amiodarone", "Vasodilators", "Co-Q 10","Warfarin", "Clopidogrel", "Folic acid", "Myocardial infarction", "Stroke", "Smoking", "Height", "Triglycerides", "Ventricular rate per minute by ECG", " Drink Beer", " Drink Wine", " Drink Liquor", "Sleep", "Creatinine urine", "Left ventricular hypertrophy", "Mitral valve", "Arrhythmia", "Dementia", "Parkinson", "Adult seizure disorder", "Neurological", "Thyroid", "Endocrine", "Renal", "Gynecologic", "Emphysema", "Pneumonia", "Asthma", "Pulmonary", "Gout", "Degenerative", "Musculoskeletal", "Gallbladder", "Gastroesophageal reflux disease", "Liver","Gastrointestinal disorders", "Hematologic disorder", "Bleeding disorder", "Eye", "Ear, Nose and throat symptoms", "Skin", "Depression", "Anxiety", "Psychosis", "Prostate", "Infectious", "Fever", "Chronic bronchitis", "COPD" be removed.

"Blood glucose", "Low-density lipoprotein", "Waist", "Weight" were removed based on their Spearman’s correlation with HFpEF.

After data pre-processing, 25 clinical characteristics were obtained ("Diuretic", "Beta blocking", "Angiotensin II antagonists"," ACEI", "Aspirin", "Coronary heart disease", "Atrial fibrillation", "Gender", "Age", "BMI", "Creatinine serum", "Average diastolic blood pressure", "Fasting blood glucose", "HDL cholesterol", "Average systolic blood pressure", "Total cholesterol", "Treated for hypertension", "Treated for lipids", "Albumin urine", "Hemoglobin a1c whole blood", "Atrial enlargement", "Rheumatic", "Aortic valve", "Rheumatoid arthritis", "C reactive protein").

## Section 2: Parameter settings of the benchmark model.

For linear SVC model, the "C" was set to "1.0", "intercept scaling" was set to "1", "loss" was set to "squared_hinge", "max iter" was set to "1000","multi_class" was set to "ovr", "penalty" was set to "l2", "random_state" was set to "None", and "tol" was set to "0.0001".

For Bagging model, the "base_estimator" was set to "None", "bootstrap" was set to "True", "bootstrap_features" was set to "False", "max_features" was set to "1.0", "max_samples" was set to "1.0", "n_estimators" was set to "10", "n_jobs" was set to "None", "oob_score" was set to "False", "random_state" was set to "0", "verbose" was set to "0", and "warm_start" was set to "False".

For Random Forest model, the "bootstrap" was set to "True", "class_weight" was set to "None", "criterion" was set to "gini", "max_depth" was set to "None", "max_features" was set to "auto", "max_leaf_nodes" was set to "None", "min_impurity_decrease" was set to "0.0", "min_impurity_split" was set to "None", "min_samples_leaf" was set to "1", "min_samples_split" was set to "2", "min_weight_fraction_leaf" was set to "0.0", "n_estimators" was set to "50", "n_jobs" was set to "-1", "oob_score" was set to "False", "random_state" was set to "0", "verbose" was set to "0", and "warm_start" was set to "False".

For RUSBoost model, the "algorithm" was set to "SAMME.R", "learning_rate" was set to "1.0", "n_estimators" was set to "10", "learning_rate" was set to "1.0", "n_estimators" was set to "20", "replacement" was set to "False", and "sampling_strategy" was set to "auto".

For EasyEnsemble mode, the "algorithm" was set to "SAMME.R", "base_estimator" was set to "None", "learning_rate" was set to "1.0", "n_estimators" was set to "10", "random_state" was set to "None", "n_estimators" was set to "10", "n_jobs" was set to "-1", "replacement" was set to "False", "sampling_strategy" was set to "auto", "verbose" was set to "0", and "warm_start" was set to "False".

For GradientBoosting model, the "criterion" was set to "friedman_mse', "init" was set to "None", "learning_rate" was set to "1.0", "loss" was set to "deviance", "max_depth" was set to "1", "max_features" was set to "None", "max_leaf_nodes" was set to "None", "min_impurity_decrease" was set to "0.0", "min_impurity_spl"it" was set to "None", "min_samples_leaf" was set to "1", "min_samples_split" was set to "2", "min_weight_fraction_leaf" was set to "0.0", "n_estimators" was set to "50", "n_iter_no_change" was set to "None", "presort" was set to "'auto", "random_state" was set to "0", "subsample" was set to "1.0", "tol" was set to "0.0001", "validation_fraction" was set to "0.1", "verbose" was set to "0", and "warm_start" was set to "False".

For XGBClassifier model, the "base_score" was set to "0.5", "booster" was set to "gbtree", "colsample_bylevel" was set to "1", "colsample_bynode" was set to "1", " colsample_bytree" was set to "0.6", "eta" was set to "0.2", "gamma" was set to "0.4", "learning_rate" was set to "0.1", "max_delta_step" was set to "0", "max_depth" was set to "6", "min_child_weight" was set to "1", "missing" was set to "None", "n_estimators" was set to "50", "n_jobs" was set to "1", "nthread" was set to "4", "objective" was set to "binary:logistic", "random_state" was set to "0", "reg_alpha" was set to "0", "reg_lambda" was set to "1", "scale_pos_weight" was set to "1", "seed" was set to "1000", "silent" was set to "1", "subsample" was set to "0.8", and "verbosity" was set to "1".

For LogitBoost model, the "base_estimator" was set to "None", "bootstrap" was set to "False", "learning_rate" was set to "1.0", "max_response" was set to "4.0", "n_estimators" was set to "150", "random_state" was set to "0", and "weight_trim_quantile" was set to "0.05".

For mixed logistic regression model, the "family" was set to "binomial", "control" was set to "glmerControl(optimizer="bobyqa")", " nAGQ " was set to "1", " devFunOnly " was set to " FALSE ", random effects terms was set to "sex".

## Section 3: Aging related CpGs in aging-related articles.

There were 71 age-related CpGs reported in the article #1[1], and we selected 26 CpGs with absolute value of the coefficient more than 10 for prediction, including "cg02650266", "cg16867657", "cg07955995", "cg20426994", "cg22285878", "cg02046143", "cg04940570", "cg06419846", "cg22213242", "cg04416734", "cg14692377", "cg21139312", "cg16054275", "cg20822990", "cg03607117", "cg00486113", "cg06685111", "cg20052760", "cg08097417", "cg14361627", "cg22796704", "cg11067179", "cg03399905", "cg09651136", "cg02867102", and "cg05442902".

Article #2[2] has 8 age-related CpGs, including "cg09462576", "cg15804973", "cg08598221", "cg26581729", "cg20654468", "cg25268718", "cg02867102", and " cg05294455".

Article #3[3] has 5 age-related CpGs including "cg02228185", "cg25809905", "cg09809672", "cg15379633", and "cg17861230".

## Section 4: “EHR+ RNA” model.

Similar to the HFmeRisk, the combined model of“EHR+ RNA” model contained a total of 761 participants did not experience heart failure and 86 HFpEF samples after eight years. For “EHR+ RNA” model, consistent with the HFmeRisk model feature selection and modeling approach, we obtained 29 clinical features and 373 differential genes after preprocessing for lasso and xgboost feature selection, and finally screened for 20 combined features, including 2 EHRs (age and urine albumin) and 18 genes (*C3AR1, FLJ45248, SLC25A44, C2CD3, COL19A1, TNP1, PLA2G12A, ZNF578, ARG1, MTRF1L, KLF10, TRIB2, TIGD1, OR10A2, C9orf30, GNG2, RGS9BP,* and *LOC92973*).

## Section 5: “EHR+ mircroRNA” model.

Similar to the HFmeRisk, the combined model of microRNA and clinical features contained a total of 776 non HFpEF and 87 HFpEF samples after eight years. For “EHR+ microRNA” model, consistent with the HFmeRisk model feature selection and modeling approach, we obtained 24 clinical features and 37 differential microRNAs after preprocessing for lasso and xgboost feature selection, and finally screened for 18 combined features, including 9 EHRs (serum creatinine, average systolic blood pressure, albumin urine, age, aortic valve, diuretic use, whole blood hemoglobin a1c, beta blocking use, and fasting blood glucose) and 9 microRNAs (miR-142-3p, miR-886-3p, miR-125b-5p, miR-186-5p-a1, miR-218-5p, miR-339-5p, miR-296-5p, miR-145-5p, and miR-128).

## Section 6: The Matthews correlation coefficient.

The Matthews correlation coefficient is used in machine learning as a measure of the quality of binary and multiclass classifications [4]. It takes into account true and false positives and negatives and is generally regarded as a balanced measure which can be used even if the classes are of very different sizes. Here is a pipeline we do the Adversarial Validation: (1) the training set and the testing set are mixed together to form a new dataset, and a new dimension label is added to the dataset, the value of which depends on the data source (training set identifier 1, testing set identifier 0) (2) Use 90% of this dataset to train the SVM model, and the remaining 10% to test the performance of the model. (3) Calculate the MCC and AUC of the model on the testing set.

## Section 7: Parameter settings of the Willliam’s model.

In Willliam model, the conditional probabilities of heart failure were estimated using a logistic function composed of 7 EHR. The regression coefficient is -9.2087(Intercept), 0.0412(age), 0.9026(electrocardiographic left ventricular hypertrophy), 0.0166(heart rate), 0.00804(systolic blood pressure), 1.6079(coronary heart disease), 0.9714(valve disease), and 0.2244(diabetes)

# Reference

1. Hannum G, Guinney J, Zhao L, Zhang L, Hughes G, Sadda S, et al. Genome-wide methylation profiles reveal quantitative views of human aging rates. Mol Cell. 2013;49(2):359-67.

2. Zhang Y, Hapala J, Brenner H, Wagner W. Individual CpG sites that are associated with age and life expectancy become hypomethylated upon aging. Clin Epigenetics. 2017;9:9.

3. Weidner CI, Lin Q, Koch CM, Eisele L, Beier F, Ziegler P, et al. Aging of blood can be tracked by DNA methylation changes at just three CpG sites. Genome Biol. 2014;15(2):R24.

4. Chicco D, Jurman G. The advantages of the Matthews correlation coefficient (MCC) over F1 score and accuracy in binary classification evaluation. BMC Genomics. 2020;21(1):6.

# Abbreviations

HFpEF: Heart failure with preserved ejection fraction; CHF: Chronic heart failure; LVEF: left ventricular ejection fraction; HFrEF: heart failure with reduced ejection fraction; HFmrEF: heart failure with intermediate ejection fraction; FHS: Framingham Heart Study; LASSO: Least Absolute Shrinkage and Selection Operator; XGBoost: Extreme Gradient Boosting; DeepFM: Factorization-Machine based neural network; CpG: cytosine-phosphate-guanine; DMPs: Differentially methylated probes; DMGs: differentially methylated genes; BMI: body mass index; AUC: area under the curve; EHR: electronic health record; MCC: Matthews correlation coefficien; UMN: University of Minnesota; JHU: Johns Hopkins University; CI: confidence interval.
