# Supplementary material for: A deep learning model for early risk prediction of heart failure with preserved ejection fraction by DNA methylation profiles combined with clinical features
Source: Clin Epigenetics. 2022 Jan 19;14:11. doi: 10.1186/s13148-022-01232-8 (PMC8772140; doi:10.1186/s13148-022-01232-8)
Supplement: Supplementary file 3 — Additional file 3. Supplementary figures. [file 13148_2022_1232_MOESM3_ESM.docx]

**Supplementary Figures**


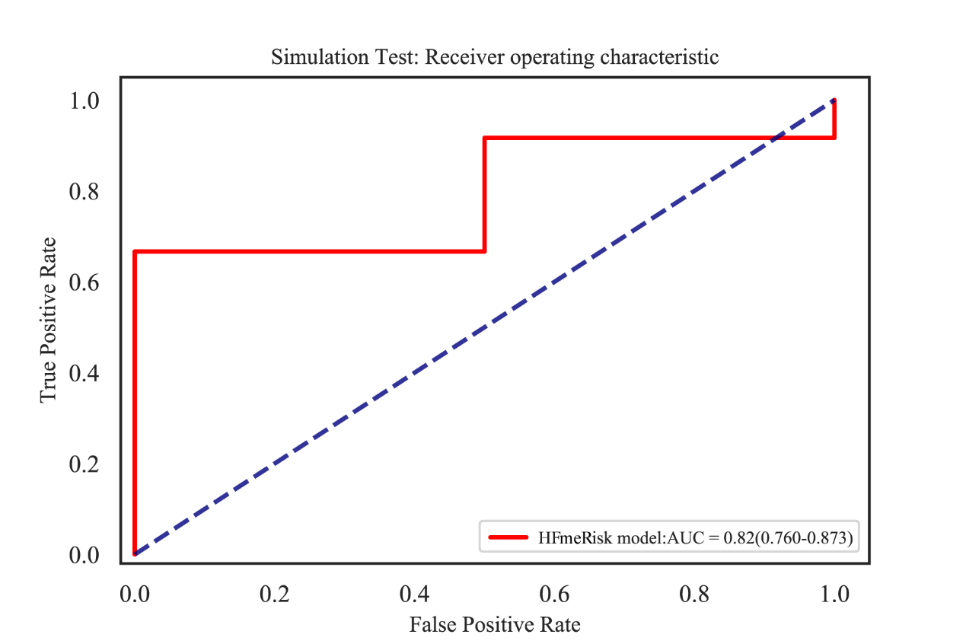


**Supplementary Figure 1. AUC results of the simulation test using HFmeRisk model.** AUC results of the HFmeRisk models in the simulation set using 30 features. AUC, Area under the ROC Curve.


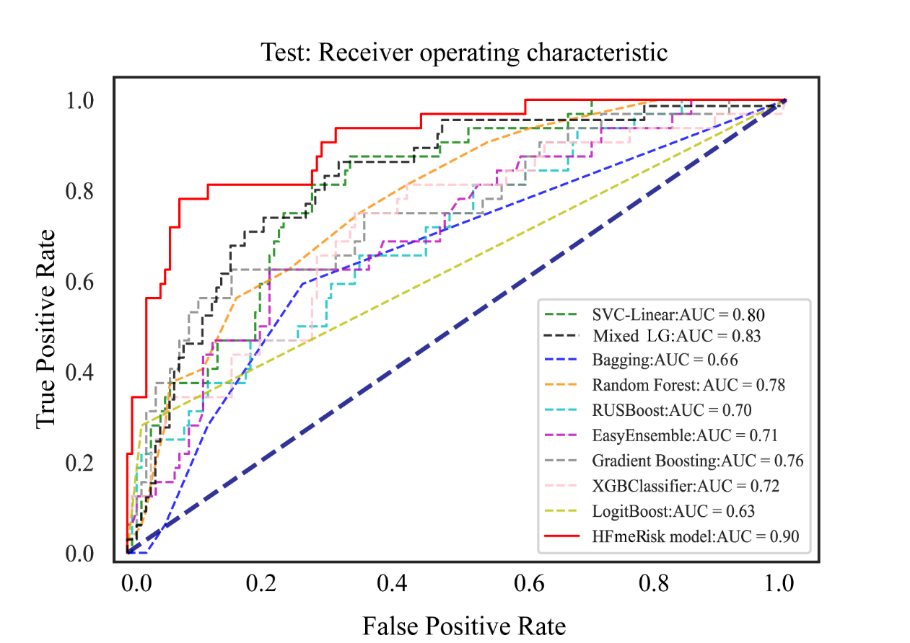


**Supplementary Figure 2. AUC results of the benchmark models.** AUC results of the benchmark models and the DeepFM model in the testing set using 30 features. AUC, Area under the ROC Curve. AUC, Area under the ROC Curve. Mixed LG, mixed logistic regression.


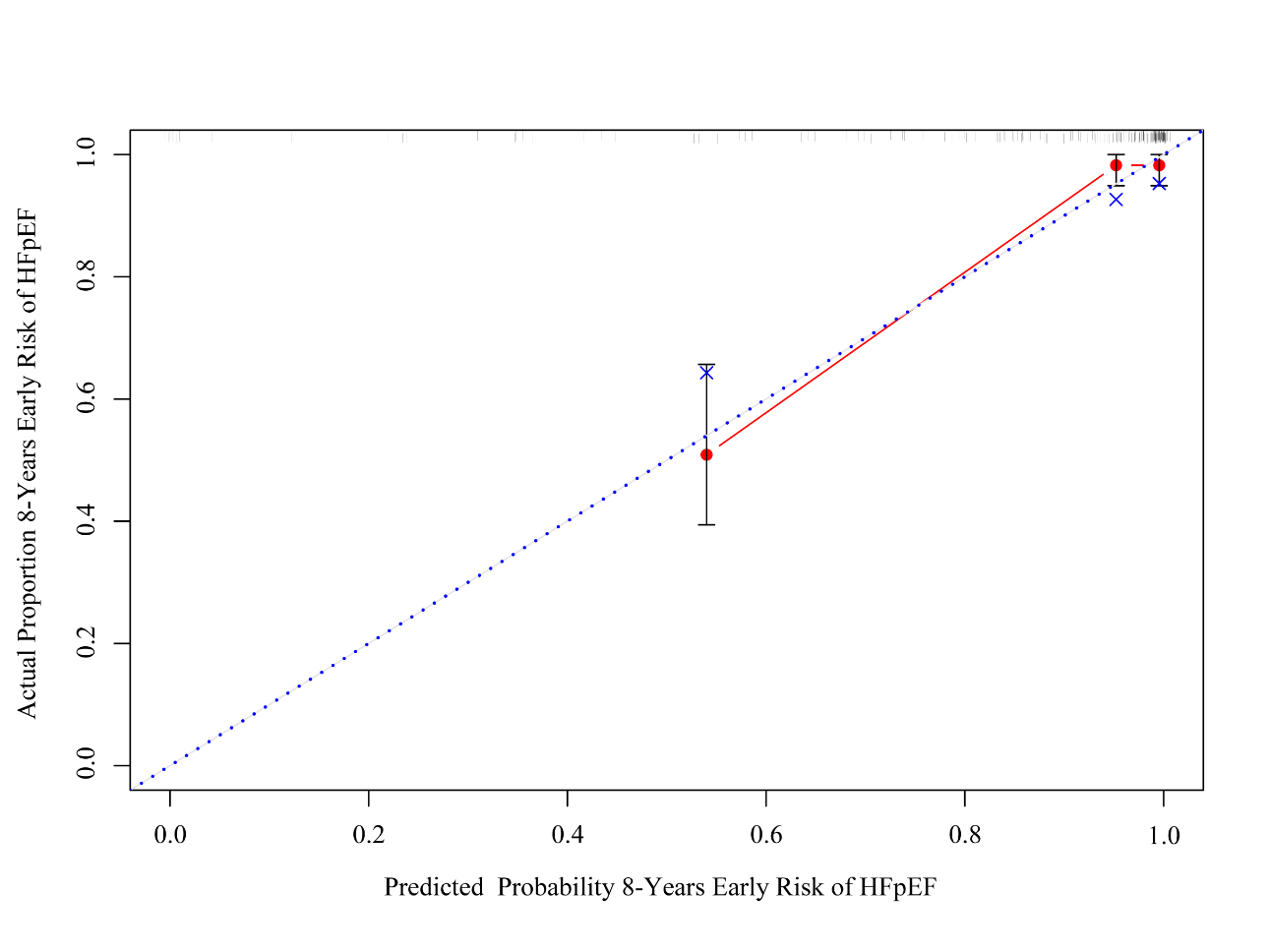


**Supplementary Figure 3. Calibration plot of the Cox model in the testing set using 30 features.** The Hosmer-Lemeshow statistic with *P* = 0.199, C statistic=0.85, where the number of bins to use to calculate quantiles is 10. HFpEF, heart failure with preserved ejection fraction.


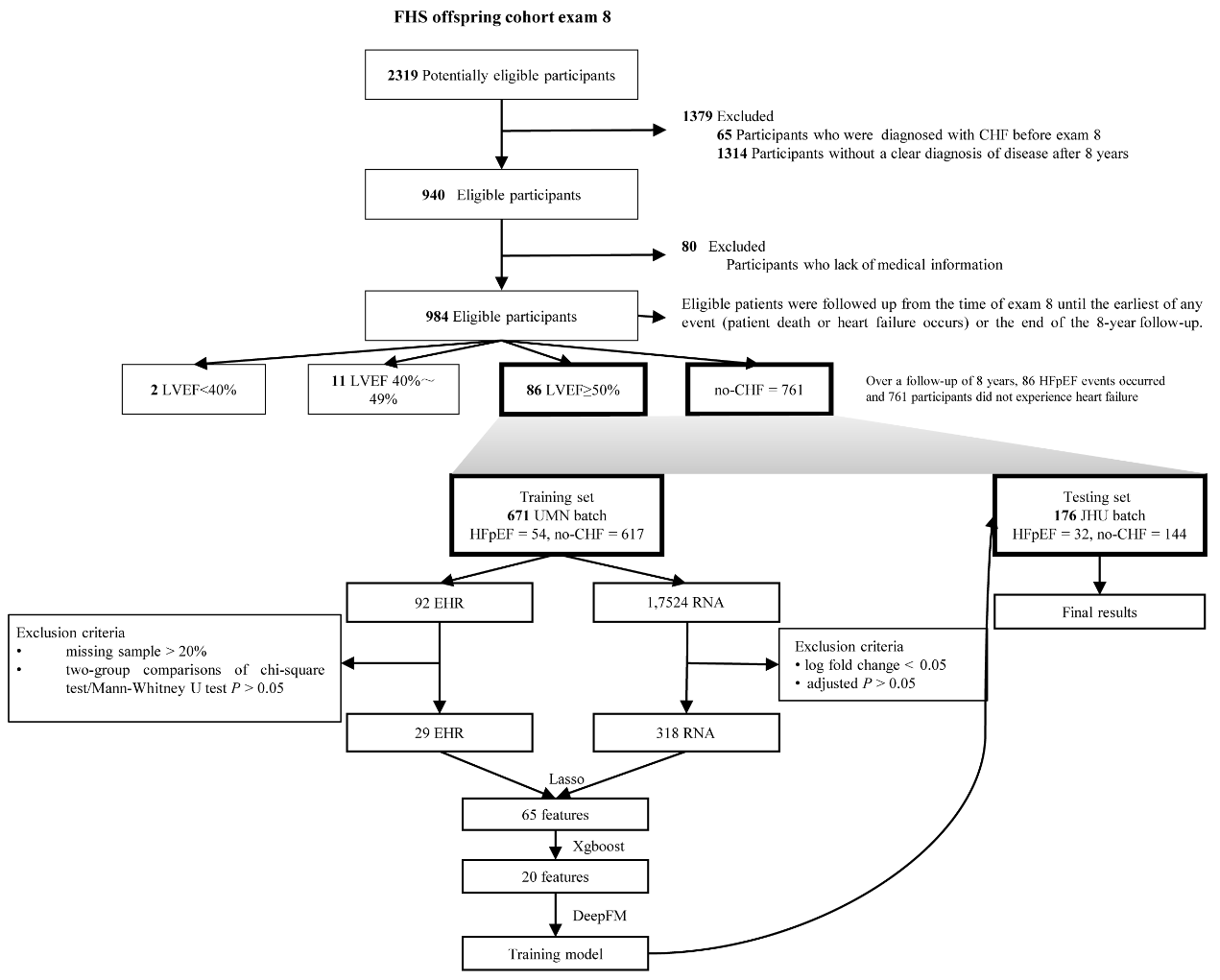


**Supplementary Figure 4. Overview of study population and study design for“EHR+ RNA” model.** FHS, Framingham Heart Study; UMN, University of Minnesota; JHU, Johns Hopkins University; CHF, chronic heart failure; LVEF, Left ventricular ejection fraction; HFpEF, heart failure with preserved ejection fraction.

**
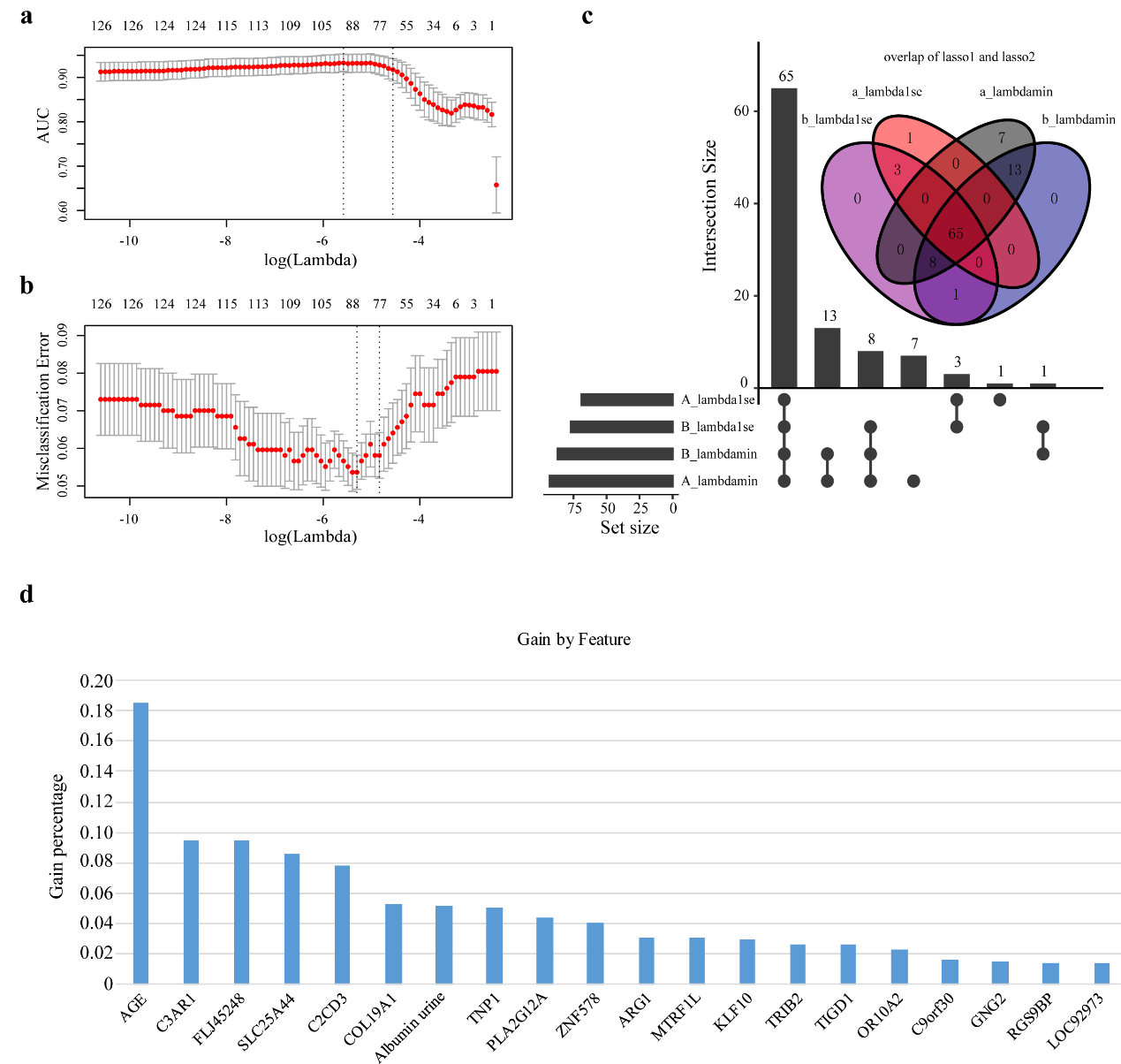
**

**Supplementary Figure 5. 20 features obtained by LASSO and XGBoost algorithms for “EHR+ RNA” model. a** AUC with different numbers characteristics as revealed by the LASSO model. AUC, Area under the ROC Curve. **b** Misclassification error for different numbers of features revealed by the LASSO model. In **a** and **b**, the grey lines represent the standard error and the vertical dotted lines represent optimal values by minimum criteria (left) and the largest value of lambda such that the error is within 1 standard error of the minimum (right). The upper abscissa is the number of non-zero coefficients in the model at this time and the lower abscissa is log Lambda, which is the tuning parameter used for 10-fold cross-validation in the LASSO model. **c** The intersection of non-zero coefficients in **a** and **b**. 65 non-zero coefficients are obtained in the LASSO model. **d** The best model features were ranked based on the gain index in xgboost model. The xgboost model further simplified the 65 features from the LASSO model, and finally, 20 valid features were obtained. The gain index represents the fractional contribution of each feature to the model based on the total gain of this feature’s splits.


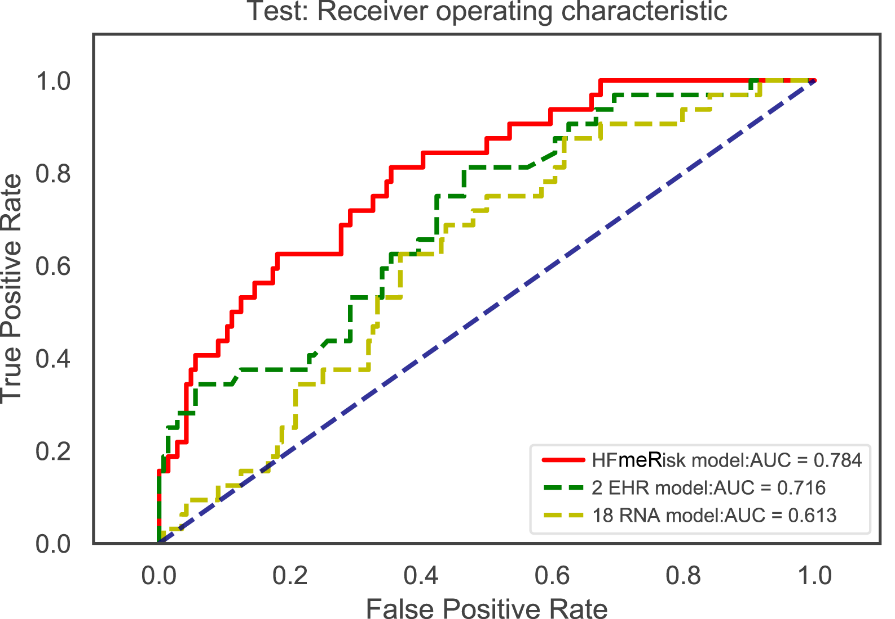


**Supplementary Figure 6. “EHR+ RNA” model results.** AUC results of the prediction performance according to different features in the testing set. “(EHR+ RNA/EHR/RNA model)” indicates the model with EHR and RNA data, the model with RNA data only, and the model with EHR data only, respectively. AUC, Area under the ROC Curve.


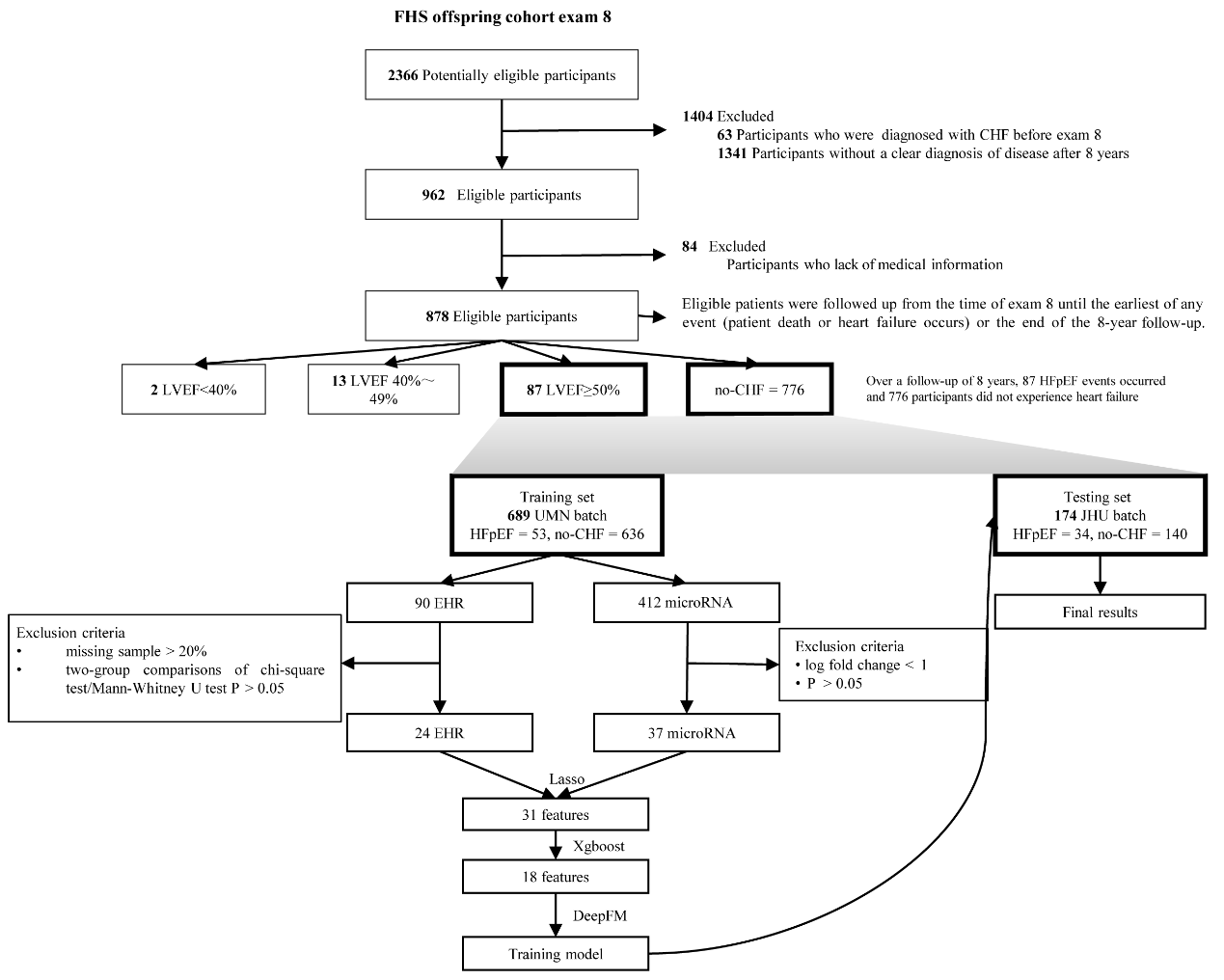


**Supplementary Figure 7. Overview of study population and study design for “EHR+ microRNA” model.** FHS, Framingham Heart Study; UMN, University of Minnesota; JHU, Johns Hopkins University; CHF, chronic heart failure; LVEF, Left ventricular ejection fraction; HFpEF, heart failure with preserved ejection fraction.

**
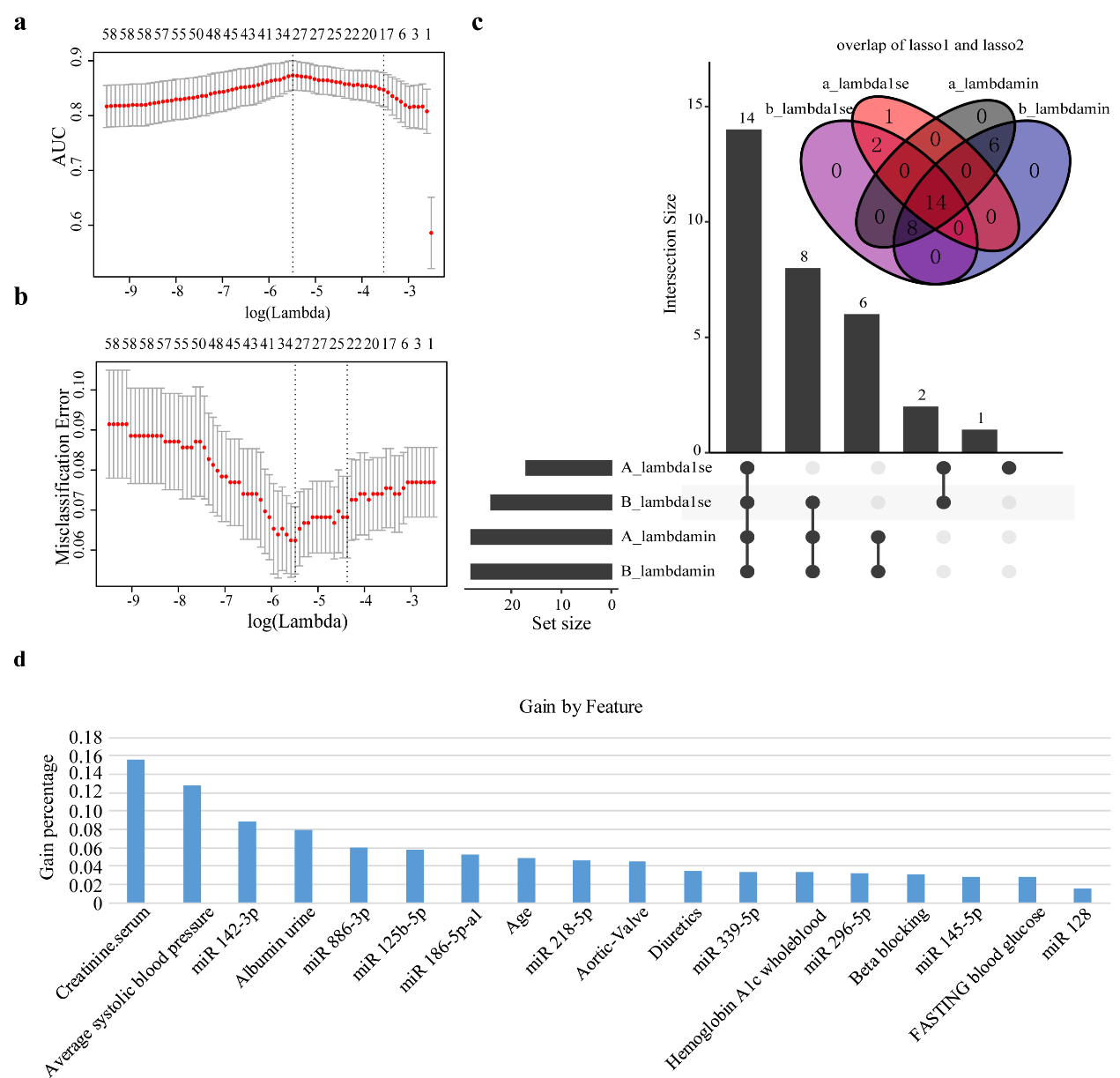
**

**Supplementary Figure 8. 18 features obtained by LASSO and XGBoost algorithms for “EHR+ microRNA” model. a** AUC with different numbers characteristics as revealed by the LASSO model. AUC, Area under the ROC Curve. **b** Misclassification error for different numbers of features revealed by the LASSO model. In **a** and **b**, the grey lines represent the standard error and the vertical dotted lines represent optimal values by minimum criteria (left) and the largest value of lambda such that the error is within 1 standard error of the minimum (right). The upper abscissa is the number of non-zero coefficients in the model at this time and the lower abscissa is log Lambda, which is the tuning parameter used for 10-fold cross-validation in the LASSO model. **c** The union of non-zero coefficients in **a** and **b**. 31 non-zero coefficients are obtained in the LASSO model. **d** The best model features were ranked based on the gain index in xgboost model. The xgboost model further simplified the 31 features from the LASSO model, and finally, 18 valid features were obtained. The gain index represents the fractional contribution of each feature to the model based on the total gain of this feature’s splits.


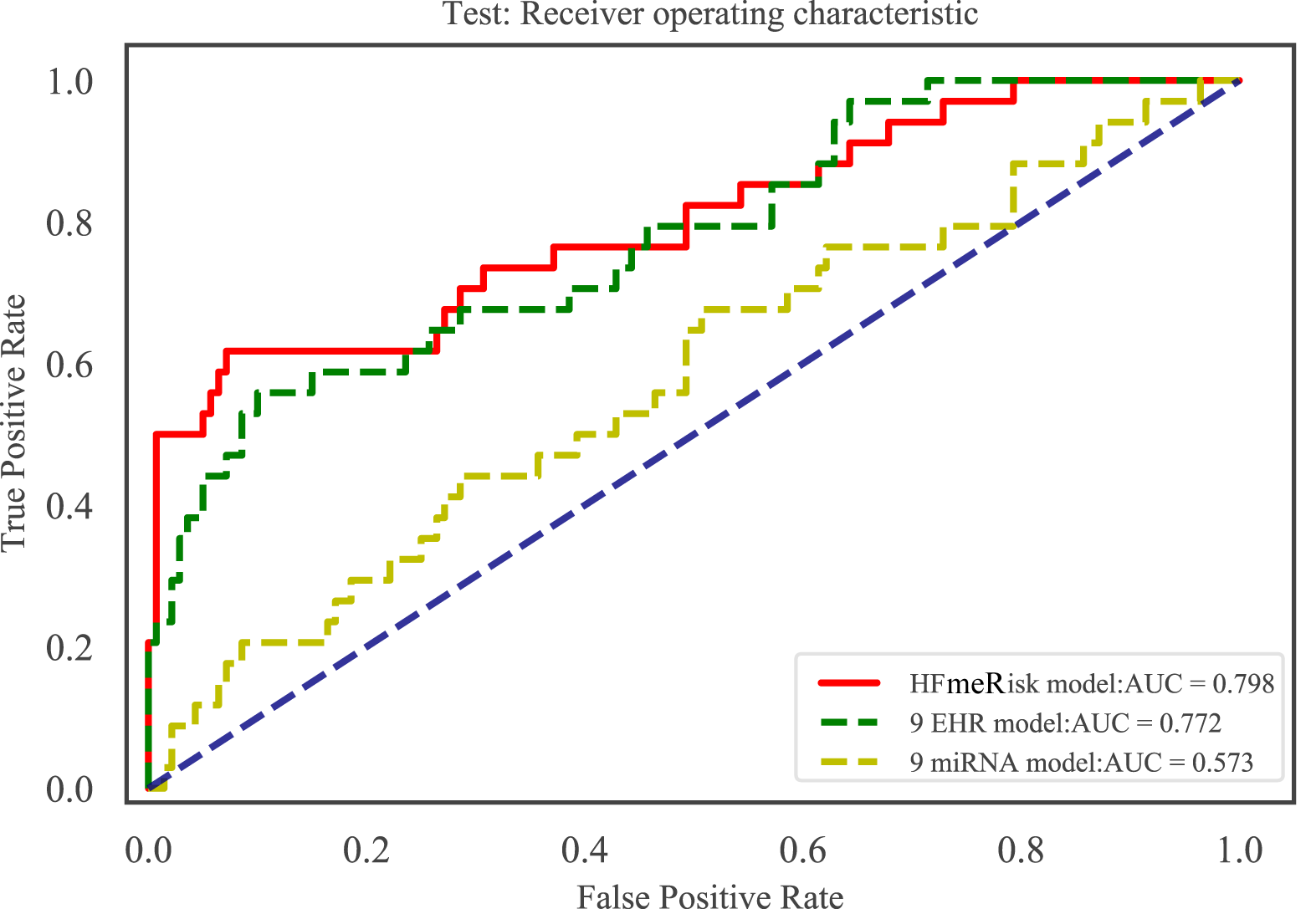


**Supplementary Figure 9. “EHR+ microRNA” model results.** AUC results of the prediction performance according to different features in the testing set. “(EHR+ microRNA/EHR/microRNA model)” indicates the model with EHR and microRNA data, the model with microRNA data only, and the model with EHR data only, respectively. AUC, Area under the ROC Curve.


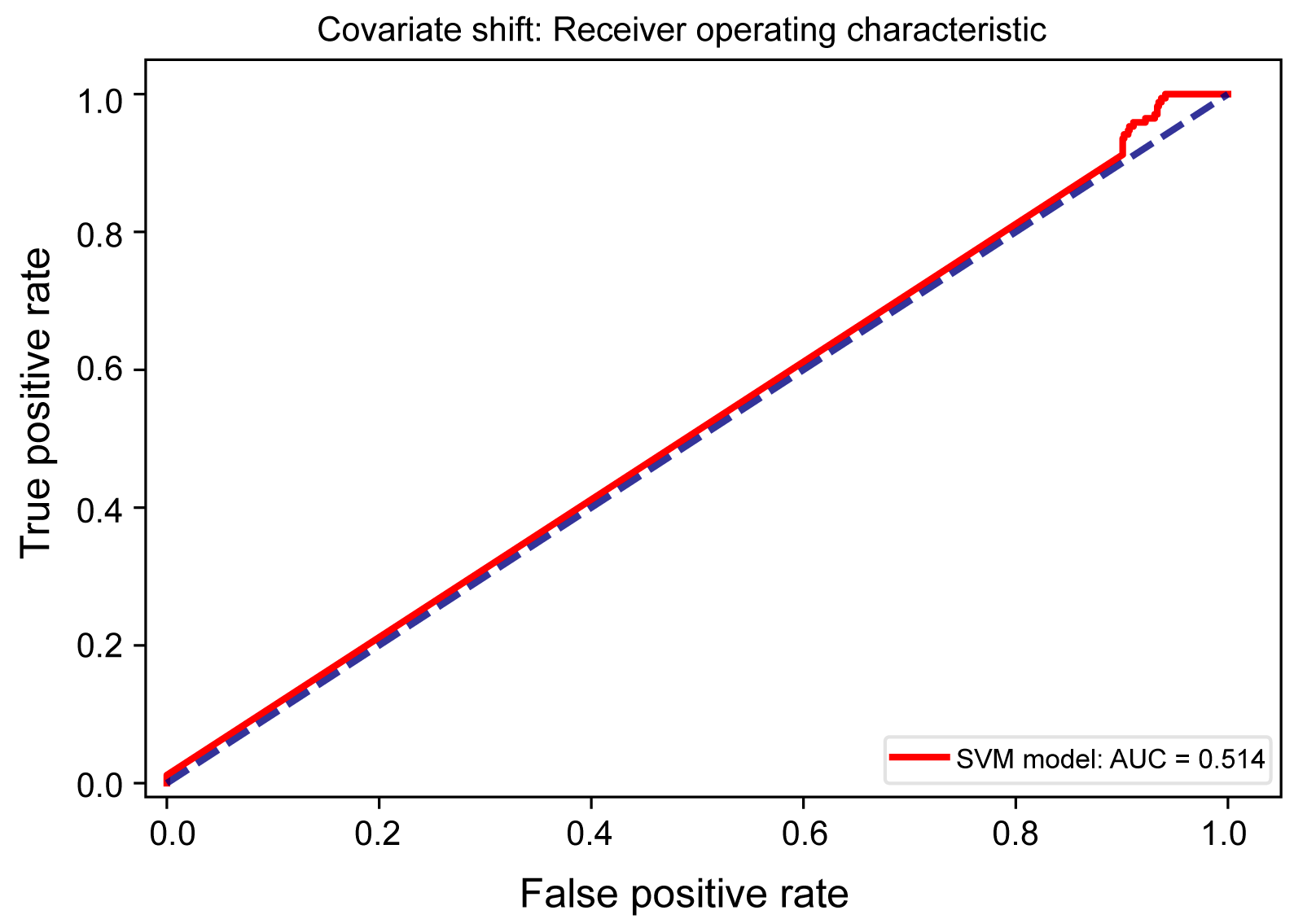


**Supplementary Figure 10. AUC result of the covariate shift test.** If a covariate shift occurs in the data, then it is theoretically possible to distinguish the training data from the testing data with a higher accuracy by a classifier.


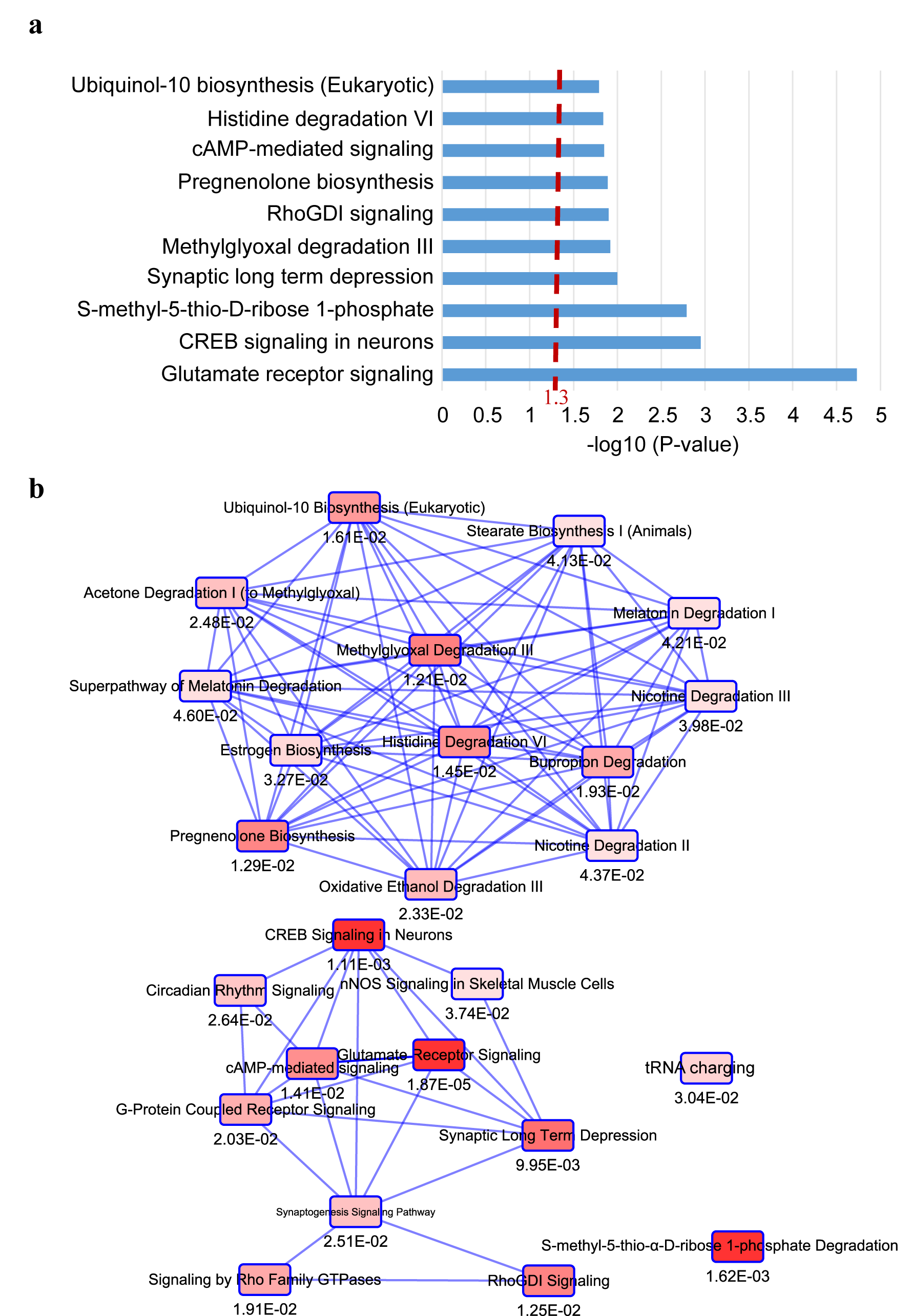


**Supplementary Figure 11. Pathway analysis on DMGs using IPA. a** Canonical pathway analysis (top 10) on DMGs using IPA. The red line is where the –log10 P-values = 1.3 (*P* = 0.05). **b** IPA-related pathways. DMGs, differentially methylated genes. DMGs, differentially methylated genes; IPA, Ingenuity Pathway Analysis.
